# Joint Entropy Error Bound of Two-Dimensional Direction-of-Arrival Estimation for L-Shaped Array

**DOI:** 10.3390/s25061929

**Published:** 2025-03-20

**Authors:** Xiaolong Kong, Daxuan Zhao, Nan Wang, Dazhuan Xu

**Affiliations:** 1 The Electronic and Information Engineering, Nanjing University of Aeronautics and Astronautics, Nanjing 210016, China; xiao_long_kong@nuaa.edu.cn (X.K.); zhaodaxuan@nuaa.edu.cn (D.Z.); n_wang@nuaa.edu.cn (N.W.); 2 The Purple Mountain Laboratories, Nanjing 211100, China

**Keywords:** direction-of-arrival estimation, joint *a posteriori* entropy, L-shaped line array, global performance bound

## Abstract

Several performance lower bounds have been studied for evaluating the accuracy of direction-of-arrival (DOA) estimation. However, lower bounds for joint estimation have not been fully explored when it comes to DOA estimation. The Cramér–Rao bound (CRB) can guarantee asymptotic tightness in the high-signal-to-noise ratio (SNR) region but cannot provide tight performance bounds for parameter estimators in low- and medium-SNR regions. Consequently, we propose a tight performance bound for the joint estimation of azimuth and elevation DOAs in an L-shaped array. Firstly, the joint conditional probability density function (PDF) is given to establish the mathematical relationship among the receiving signal and the azimuth and elevation DOAs. Then, the joint a posteriori PDF is derived according to the Bayesian theorem. Next, the azimuth and elevation DOA entropy error bound (AEEEB) is derived as a global performance bound using the joint *a posteriori* entropy. Finally, the CRB and the mean square error (MSE) are provided for comparisons with the proposed performance bound. The simulation results indicate that the AEEEB provides a tighter performance bound compared to the CRB.

## 1. Introduction

As global traffic volume continues to rise, traffic safety has become a focal point of societal concern. According to reports from the World Health Organization, traffic accidents result in hundreds of thousands of casualties annually, underscoring the urgency of addressing traffic safety issues. To effectively reduce the incidence of traffic accidents, the development and application of Advanced Driver-Assistance Systems (ADASs) and autonomous driving technologies are particularly crucial. In this context, direction-of-arrival (DOA) estimation, as a key signal processing technique, is increasingly recognized as an important means to enhance traffic safety. Traditional DOA estimation methods are often limited to one-dimensional (1D) scenarios, primarily focusing on the azimuth angle of signal sources, which may not provide enough information in complex traffic environments [1,2,3]. In contrast, two-dimensional (2D) DOA estimation technology can capture both azimuth and elevation angles, offering vehicles a comprehensive environmental awareness capability [4]. By integrating sensor arrays and relevant signal processing algorithms, 2D DOA estimation can effectively identify various signal sources, including surrounding vehicles, pedestrians, traffic signs, and traffic lights, which is crucial for enhancing the vehicle’s intelligent decision-making abilities. In practical applications, 2D DOA estimation can significantly improve early warning capabilities for traffic accidents [5,6]. For instance, by continuously monitoring the surrounding environment, the system can detect potential collision risks and take appropriate actions, such as automatic braking or issuing alerts, thereby effectively reducing the likelihood of accidents. Furthermore, this technology can also be employed for traffic flow analysis and management, enhancing road utilization efficiency and further ensuring traffic safety.

**Figure 1:**
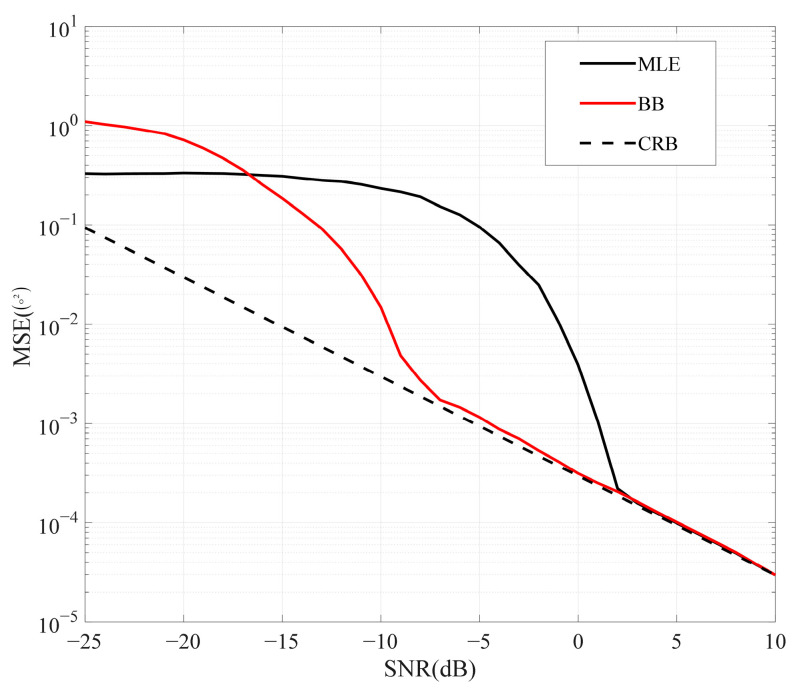
The performance of MLE, the BB, and the CRB versus SNR (DOA estimation in uniform line array).

Two-dimensional DOA estimation is a fundamental problem in array signal processing and serves as a key metric for evaluating the performance of automotive sensors [7,8]. The 2D DOA estimation technique holds great potential for applications in signal processing and communication, particularly in fields such as radar, sonar, and wireless communication. However, despite the increasing maturity of its theoretical foundation, its estimation performance in practical applications is often influenced by various factors, primarily including the strength of noise, the number of array elements, and environmental conditions. Therefore, establishing a tight and reliable performance bound for 2D DOA estimation is vital for ensuring the effectiveness of traffic safety systems [9]. Error is a critical metric for evaluating the performance of parameter estimation methods, with the most representative metric being the mean squared error (MSE), expressed as

(1) $$\mathrm{MSE}=E_{\theta }\left[{\left(\theta -\theta_{0}\right)}^{2}\right]$$

where θ represents the estimated value, and $\theta_{0}$ represents the true value. Due to its advantages of ease of calculation and intuitiveness, the MSE has been widely applied over the past half-century [10,11]. However, its drawback lies in the lack of an exact explicit minimum solution. Consequently, researchers have extensively explored the performance bounds of parameter estimation. Most studies have focused on the Cramér–Rao bound (CRB) and Barankin bound (BB), and analytical expressions are successfully derived for various scenarios [12,13,14,15]. The CRB is widely accepted as a performance lower bound in parameter estimation due to its easily derivable explicit solution. In [16], the CRB of direction of arrival (DOA) is derived for coprime arrays. Although the CRB provides a dependable and stringent performance bound in the high-signal-to-noise ratio (SNR) region, its performance is catastrophic in the low-SNR region. Compared to the CRB, the BB has tighter performance in the medium-SNR region. However, the BB completely diverges from the CRB in the low-SNR region, exhibiting a phenomenon known as the threshold effect. The estimation performances of the CRB, the BB, and the MSE of the maximum likelihood estimation (MLE) method are plotted in Figure 1. In the high-SNR region, both the CRB and the BB can accurately predict the performance of the MLE. However, in the low-SNR region, it is evident that the BB surpasses the MLE in terms of the MSE, and the CRB cannot provide accurate performance in parameter estimation. The occurrence of this phenomenon, known as the threshold effect, is due to the lack of *a priori* distribution of parameters in the low-SNR region. A similar phenomenon can also be seen in [17]. Therefore, neither the CRB nor the BB can fully characterize performance across all SNR regions.

The rapid advancement of signal processing techniques has pushed parameter estimation in the low-to-medium-SNR region to the forefront of research. Consequently, while the CRB provides theoretical guidance, analyzing the accuracy of parameter estimation using it often faces challenges. It is recognized that the convergence of the MSE is affected by the *a priori* distribution of parameters in the extremely low SNR region. Hence, Bayesian bounds have been introduced in recent years. Bayesian bounds, such as Weiss–Weinstein bound (WWB) and the Ziv–Zakai bound (ZZB), leverage the *a priori* distribution of parameters to establish a global performance bound, enhancing the performance of parameter estimation in the low-to-medium-SNR region [18,19,20,21]. The contributions of the ZZB and the WWB to parameter estimation performance have also been demonstrated in many studies. The explicit WWB is derived for multiple-input multiple-output (MIMO) radar with collocated linear arrays [22]. In [23], the explicit ZZB is derived for DOA estimation. By introducing the *a priori* distribution of the parameter, the ZZB and the WWB provide a rigorous and reliable performance bound for parameter estimation in all SNR regions. However, the main drawback of the two performance bounds is their high computational complexity. WWB calculation necessitates the establishment of test points and the inversion of a matrix, while computation for the ZZB involves performing multiple complex numerical integrals [24].

Unlike the ZZB and the WWB, the entropy error bound (EEB) provides a global performance bound by utilizing the *a posteriori* entropy of the parameters. The EEB is a globally strict and tight performance bound. It possesses a simple comprehensive expression, which is a function of array parameters such as the SNR, array elements, and the ratio of signal bandwidth to carrier frequency. The expression for the EEB has been derived for various array scenarios. For example, in [25], the mutual information bound is derived in a single-source 1D DOA estimation scenario, allowing for the computation of the EEB. In [26], the EEB is proposed to evaluate DOA estimation in a compressive sensing scenario. The results indicate that the EEB can provide tight and rigorous performance lower bounds in the whole SNR range.

In the context of complex application scenarios, it is crucial to establish performance bounds for 2D DOA estimation. Two-dimensional DOA estimation requires precise measurement of both azimuth and elevation angles. During this transition, the analysis of performance bounds becomes more complex yet remains equally important. Our previous research on sensor array DOA estimation primarily focused on deriving a closed-form expression for the 1D EEB. We shift our focus to 2D joint estimation, marking a significant advancement in the parameter estimation field. Two-dimensional DOA estimation incorporates both azimuth and elevation angles, offering a more comprehensive signal analysis capability. This transition enables us to better address complex signal environments. Therefore, the focus of this study is to derive a joint azimuth and elevation DOA EEB (AEEEB) in a single-signal-source scenario. Assuming the azimuth and elevation DOAs follow a uniform distribution within the observation interval, we obtained the *a posteriori* probability density function (PDF) of azimuth and elevation DOAs via the Bayesian theorem. By calculating the *a posteriori* distribution uncertainty of azimuth and elevation DOAs, known as *a posteriori* entropy, we provide the theoretical AEEEB. Furthermore, by introducing the parameter uncertainty factor $p_{s}$ into the theoretical AEEEB, we derive a global joint AEEEB. The main highlights of this paper are as follows:

First, assuming that the *a priori* distributions of azimuth and elevation DOAs follow a uniform distribution, we derive the joint *a posteriori* PDF for the azimuth and elevation DOAs. Subsequently, we demonstrate that the joint *a posteriori* PDF approximately follows a 2D Gaussian distribution in the high-SNR region.

Then, based on the joint *a posteriori* PDF, we derive the theoretical AEEEB, which is an effective performance bound in all SNR regions. We also give the joint AEEEB by introducing the parameter uncertainty factor. The simulation results demonstrate that the approximate AEEEB remains consistent with the theoretical AEEEB in all SNR regions.

Next, we also consider the scenario of independently evaluating the estimation performance of azimuth and elevation DOAs, and the expressions for the azimuth EEB (AEEB) and elevation EEB (EEEB) are derived. And we demonstrate that the AEEEB, the AEEB, and the EEEB degenerate to the respective CRBs in their respective scenarios under the high-SNR condition.

Finally, we find that the joint expressions for the AEEB, the EEEB, and the AEEEEB are a function of array system parameters. Specifically, the three performance bounds are highly influenced by the observation interval in the low-SNR region. In the high-SNR region, the three performance bounds depend on the number of array elements and the SNR.

The symbols employed throughout this paper are summarized in Table 1. In addition, the bold uppercase letters represent matrices, and the bold lowercase letters represent vectors. The remainder of this manuscript is organized as follows: In Section 2, the system model is briefly presented. In Section 3, the joint *a posteriori* PDF is derived for azimuth and elevation DOAs. In Section 4, the theoretical AEEEB and the joint AEEEB are derived. In Section 5, extensive simulation results are presented to demonstrate the performance of the derived AEEEB. Finally, conclusions are drawn in Section 6.

## 2. System Model

**Figure 2:**
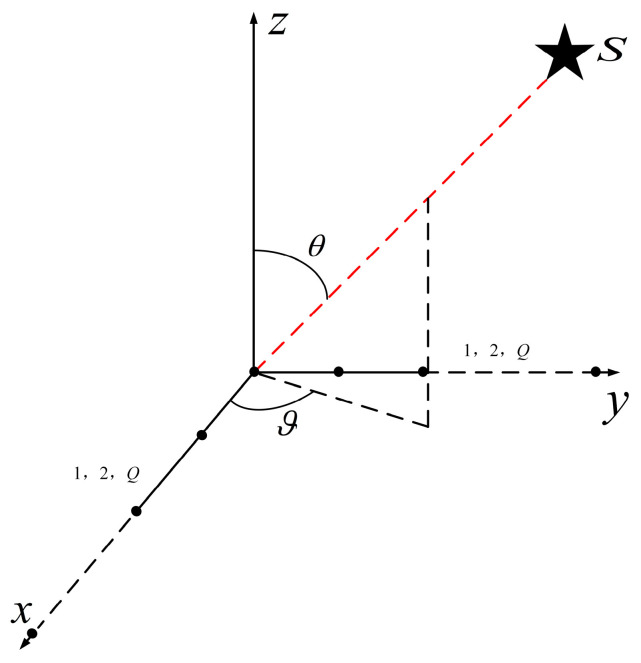
The L-shaped array system (single-signal-source scenario).

As shown in Figure 2, an L-shaped array with *Q* elements on both the *x*-axis and the *y*-axis is arranged, with a spacing of *d* between elements [27,28]. Consider the following scenario, where a far-field narrowband signal source is incident on the L-shaped array system, with an azimuth ϑ and an elevation θ.

The received signal $Z_{x}\in {\mathbb{C}}^{Q\times 1}$ of the *x*-axis is expressed as

(2) $$Z_{x}\;=\;A_{x}\left(\vartheta ,\theta \right)s\;+\;N_{x},$$

where s represents the complex scattering property for the far-field narrowband signal source, $N_{x}\in {\mathbb{C}}^{Q\times 1}$ represents the received noise matrix, and $A_{x}\left(\vartheta ,\theta \right)\in {\mathbb{C}}^{Q\times 1}$ represents the steering vector matrix, and it is written as

(3) $$A_{x}\left(\vartheta ,\theta \right)=\left[\begin{array}{c} 1\\ e^{\frac{j2\pi d\cos\vartheta \sin\theta }{\lambda }}\\ \vdots \\ e^{\frac{j2\pi (Q-1)d\cos\vartheta \sin\theta }{\lambda }} \end{array}\right].$$

The received signal $Z_{y}\in {\mathbb{C}}^{Q\times 1}$ of the *y*-axis is expressed as

(4) $$Z_{y}\;=\;A_{y}\left(\vartheta ,\theta \right)s\;+\;N_{y},$$

where $N_{y}\in {\mathbb{C}}^{Q\times 1}$ represents the received noise matrix, and $A_{y}\left(\vartheta ,\theta \right)\in {\mathbb{C}}^{Q\times 1}$ represents the steering vector matrix, and it is written as

(5) $$A_{y}\left(\vartheta ,\theta \right)=\left[\begin{array}{c} 1\\ e^{\frac{j2\pi d\sin\vartheta \sin\theta }{\lambda }}\\ \vdots \\ e^{\frac{j2\pi (Q-1)d\sin\vartheta \sin\theta }{\lambda }} \end{array}\right].$$

Finally, the received signal of the L-shaped array system can be written as

(6) $$Z=\left[\begin{array}{c} Z_{x}\\ Z_{y} \end{array}\right]=\left[\begin{array}{c} A_{x}\left(\vartheta ,\theta \right)\\ A_{y}\left(\vartheta ,\theta \right) \end{array}\right]S+\left[\begin{array}{c} N_{x}\\ N_{y} \end{array}\right]=A\left(\vartheta ,\theta \right)S+N.$$

where $Z\in {\mathbb{C}}^{Q\times 2}$ represents the received signal of the L-shaped array, $A\left(\vartheta ,\theta \right)\in {\mathbb{C}}^{Q\times 2}$ represents the steering vector matrix of the L-shaped array, and $N\in {\mathbb{C}}^{Q\times 2}$ represents the noise of the L-shaped array. The number of array elements Q will be provided in the simulation tests in Section 5.

Then, we make the assumptions outlined below regarding the signal source and array system.

**Assumption** **1.** *The signal has a bandwidth much smaller than its center frequency, i.e.,*

(7) $$B_{w}/f_{0}<1/10,$$

*where* $B_{w}$ *is the bandwidth of the signal, and* $f_{0}$ *is the center frequency of the signal.*

**Assumption** **2.** *The parameters of the signal, such as azimuth and elevation DOAs, follow a uniform distribution within the observation interval (for the parameters to be estimated, it is commonly assumed that the a priori distribution follows a uniform distribution, such as in [9,19,24]), i.e.,*

(8) $$p\left(\theta \right)=\frac{1}{\left|\Theta \right|},$$

*where* $\left|\Theta \right|$ *represents the length of the observation interval.*

(9) $$p\left(\vartheta \right)=\frac{1}{\left|\Xi \right|},$$

*where* $\left|\Xi \right|$ *represents the length of the observation interval.*

**Assumption** **3.** *We choose the Swerling 0 model as the complex scattering property of the signal source, i.e.,*

(10) $$s=\alpha e^{j\varphi }$$

*where* α *denotes the complex scattering coefficient, and* φ *denotes the initial phase and follows a uniform distribution within the observation interval, i.e.,*

(11) $$p\left(\varphi \right)=\frac{1}{2\pi }.$$

**Assumption** **4.** *In the subsequent analysis, we consider a single-source scenario. The received signal is expressed as* $Z\;=\;A\left(\vartheta ,\theta \right)S\;+\;N$*. Meanwhile, the system noise is the complex additive white Gaussian noise (CAWGN) with zero-mean and the same power on each element, with the PDF expressed as*

(12) $$p\left(N\right)={\left(\frac{1}{\pi N_{0}}\right)}^{Q^{2}}\exp\left(\frac{1}{N_{0}}{\left\|N\right\|}_{2}^{2}\right),$$

*where* $N_{0}$ *denotes the power spectral density of the noise.*

## 3. Joint *a Posteriori* Probability Density Function for Azimuth and Elevation DOAs

Consider substituting (6) into (12); we have

(13) $$p\left(z\left|\vartheta ,\theta ,\varphi \right.\right)={\left(\frac{1}{\pi N_{0}}\right)}^{Q^{2}}\exp\left(\frac{1}{N_{0}}{\left\|z-A\left(\vartheta ,\theta \right)\alpha e^{j\varphi }\right\|}_{2}^{2}\right).$$

(13) is based on a conditional PDF, which links the received signal, the azimuth, the elevation, and the initial phase. Since the phase φ is uniformly distributed within the observation interval, it follows

(14) $$\begin{array}{ll} p\left(z\left|\vartheta ,\theta \right.\right) & =\int_{0}^{2\pi }\pi \left(\varphi \right)p\left(z\left|\vartheta ,\theta ,\varphi \right.\right)d\varphi \\ & =\frac{1}{2\pi }{\left(\frac{1}{\pi N_{0}}\right)}^{Q^{2}}\int_{0}^{2\pi }\exp\left(\frac{1}{N_{0}}{\left\|z-A\left(\vartheta ,\theta \right)\alpha e^{j\varphi }\right\|}_{2}^{2}\right)d\varphi . \end{array}$$

Upon further simplification, (14) can be reduced to

(15) $$\begin{array}{ll} p\left(z\left|\vartheta ,\theta \right.\right) & =\frac{1}{2\pi }{\left(\frac{1}{\pi N_{0}}\right)}^{Q^{2}}\exp\left(\frac{1}{N_{0}}{\left\|z+\alpha^{2}\right\|}_{2}^{2}\right)•\int_{0}^{2\pi }\exp\left(\frac{2\alpha }{N_{0}}\left|e^{-j\varphi }A^{H}\left(\vartheta ,\theta \right)z\right|\right)d\varphi \\ & ={\left(\frac{1}{\pi N_{0}}\right)}^{Q^{2}}\exp\left(\frac{1}{N_{0}}{\left\|z+\alpha^{2}\right\|}_{2}^{2}\right)•I_{0}\left(\frac{2\alpha }{N_{0}}\left|A^{H}\left(\vartheta ,\theta \right)z\right|\right), \end{array}$$

where $I_{0}\left(x\right)$ represents the zero-order first-kind Bessel function [26], which is written as

(16) 12π∫02πexp2αN0e−jφAHϑ,θzdφ=12π∫02πexp2αN0ReAHϑ,θzcosφ+ImAHϑ,θzsinφdφ=I02αN0AHϑ,θz22.

According to the Bayesian theorem, we describe the relationship between the received data and unknown parameters using a joint probability distribution [29,30]. Thus, based on (8), (9), and (15), the joint *a posteriori* PDF for azimuth and elevation DOAs is calculated as

(17) $$\begin{array}{ll} p\left(\vartheta ,\theta \left|z\right.\right) & =\frac{p\left(\theta \right)p\left(\vartheta \right)p\left(z\left|\vartheta ,\theta \right.\right)}{\int_{0}^{\left|\Xi \right|}\int_{0}^{\left|\Theta \right|}p\left(\theta \right)p\left(\vartheta \right)p\left(z\left|\vartheta ,\theta \right.\right)d\vartheta d\theta }\\ & =\frac{\frac{1}{\left|\Xi \right|}\frac{1}{2\pi }{\left(\frac{1}{\pi N_{0}}\right)}^{Q^{2}}\exp\left(\frac{1}{N_{0}}{\left\|z+\alpha^{2}\right\|}_{2}^{2}\right)•I_{0}\left(\frac{2\alpha }{N_{0}}\left|A^{H}\left(\vartheta ,\theta \right)z\right|\right)}{\int_{0}^{\left|\Xi \right|}\int_{0}^{\left|\Theta \right|}\frac{1}{\left|\Xi \right|}\frac{1}{2\pi }{\left(\frac{1}{\pi N_{0}}\right)}^{Q^{2}}\exp\left(\frac{1}{N_{0}}{\left\|z+\alpha^{2}\right\|}_{2}^{2}\right)•I_{0}\left(\frac{2\alpha }{N_{0}}\left|A^{H}\left(\vartheta ,\theta \right)z\right|\right)d\vartheta d\theta }\\ & =\frac{I_{0}\left(\frac{2\alpha }{N_{0}}\left|A^{H}\left(\vartheta ,\theta \right)z\right|\right)}{\int_{0}^{\left|\Xi \right|}\int_{0}^{\left|\Theta \right|}I_{0}\left(\frac{2\alpha }{N_{0}}\left|A^{H}\left(\vartheta ,\theta \right)z\right|\right)d\vartheta d\theta }, \end{array}$$

where the denominator represents the normalization constant, and the numerator determines the shape of the *a posteriori* probability distribution.

Assume that the signal source is located at $\left(\vartheta_{0},\theta_{0}\right)$. Substituting the known received signal into (17), we have

(18) $$\begin{array}{ll} p\left(\vartheta ,\theta \left|z\right.\right) & =\frac{I_{0}\left(\frac{2\alpha }{N_{0}}\left|A^{H}\left(\vartheta ,\theta \right)\left(A\left(\vartheta_{0},\theta_{0}\right)S+N\right)\right|\right)}{\int_{0}^{\left|\Xi \right|}\int_{0}^{\left|\Theta \right|}I_{0}\left(\frac{2\alpha }{N_{0}}\left|A^{H}\left(\vartheta ,\theta \right)\left(A\left(\vartheta_{0},\theta_{0}\right)S+N\right)\right|\right)d\vartheta d\theta }\\ & =\frac{I_{0}\left(\frac{2\alpha }{N_{0}}\left|A^{H}\left(\vartheta ,\theta \right)A\left(\vartheta_{0},\theta_{0}\right)\alpha e^{j\varphi_{0}}+A^{H}\left(\vartheta ,\theta \right)N\right|\right)}{\int_{0}^{\left|\Xi \right|}\int_{0}^{\left|\Theta \right|}I_{0}\left(\frac{2\alpha }{N_{0}}\left|A^{H}\left(\vartheta ,\theta \right)A\left(\vartheta_{0},\theta_{0}\right)\alpha e^{j\varphi_{0}}+A^{H}\left(\vartheta ,\theta \right)N\right|\right)d\vartheta d\theta }\\ & =\frac{I_{0}\left(2\rho^{2}\left|G\left(\vartheta ,\theta \right)+\frac{1}{\alpha }F\left(\vartheta ,\theta ,N\right)\right|\right)}{\int_{0}^{\left|\Xi \right|}\int_{0}^{\left|\Theta \right|}I_{0}\left(2\rho^{2}\left|G\left(\vartheta ,\theta \right)+\frac{1}{\alpha }F\left(\vartheta ,\theta ,N\right)\right|\right)d\vartheta d\theta } \end{array}$$

where $\rho^{2}=\alpha^{2}/N_{0}$ represents the SNR of the antenna array.

$G\left(\vartheta ,\theta \right)$ is referred to as the steering vector of the antenna array and is expressed as

(19) $$\begin{array}{ll} G\left(\vartheta ,\theta \right) & ==\sum_{q=1}^{Q}e^{j2\pi qd\left(\cos\vartheta \sin\theta -\cos\vartheta_{0}\sin\theta_{0}\right)/\lambda }+\sum_{q=1}^{Q}e^{j2\pi qd\left(\sin\vartheta \sin\theta -\sin\vartheta_{0}\sin\theta_{0}\right)/\lambda }\\ & =G_{x}\left(\vartheta ,\theta \right)+G_{y}\left(\vartheta ,\theta \right), \end{array}$$

where

(20) $$G_{x}\left(\vartheta ,\theta \right)=Qe^{j\pi \left(Q-1\right)d\left(\cos\vartheta \sin\theta -\cos\vartheta_{0}\sin\theta_{0}\right)/\lambda }\cdot \frac{\sin\left(\pi Qd\left(\cos\vartheta \sin\theta -\cos\vartheta_{0}\sin\theta_{0}\right)/\lambda \right)}{Q\sin\left(\pi d\left(\cos\vartheta \sin\theta -\cos\vartheta_{0}\sin\theta_{0}\right)/\lambda \right)},$$

(21) $$G_{y}\left(\vartheta ,\theta \right)=Qe^{j\pi \left(Q-1\right)d\left(\sin\vartheta \sin\theta -\sin\vartheta_{0}\sin\theta_{0}\right)/\lambda }\cdot \frac{\sin\left(\pi Qd\left(\sin\vartheta \sin\theta -\sin\vartheta_{0}\sin\theta_{0}\right)/\lambda \right)}{Q\sin\left(\pi d\left(\sin\vartheta \sin\theta -\sin\vartheta_{0}\sin\theta_{0}\right)/\lambda \right)}.$$

$F\left(\vartheta ,\theta ,w\right)$ is the cross-correlation function between the received signal and noise, representing the influence of noise.

**Figure 3:**
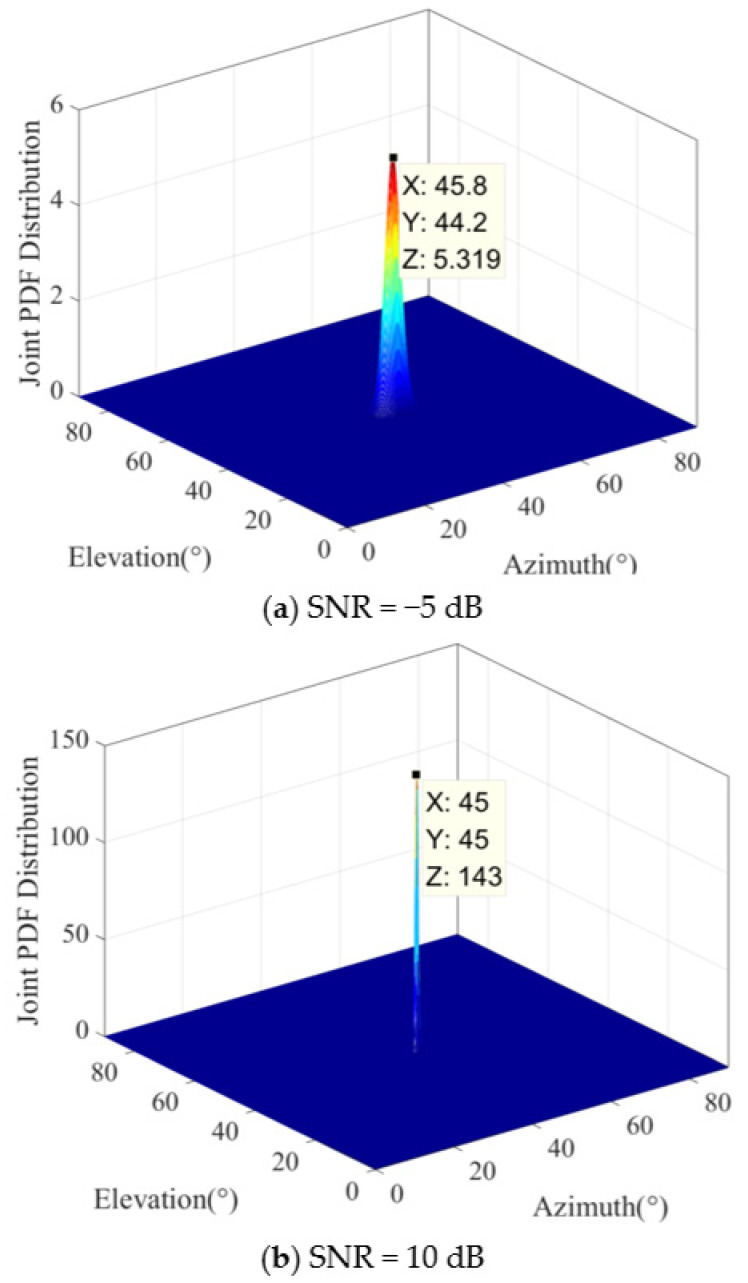
The shape of the *a posteriori* distribution when the signal source is located at $\left(45^{{}^{\circ}},45^{{}^{\circ}}\right)$. Antenna array elements: *M* = 16; antenna array spacing: *d* = 1.

The shape of the *a posteriori* distribution under different SNR conditions is shown in Figure 3 when the signal source is located at (45°, 45°). The color variations in the figure represent changes in the posterior probability density function values, with blue indicating the minimum value and red indicating the maximum value. In the low-SNR region, the system noise has a significant impact on the received signal, while the signal from the source is relatively weak. The *a posteriori* distribution of the azimuth and elevation DOA joint estimation is typically more spread out and blurred, exhibiting a wider range of values. In this case, the estimation results may have greater uncertainty, as shown in Figure 3a. Contrary to the low-SNR situation, the influence of noise is relatively small under high-SNR conditions. Therefore, the *a posteriori* distribution of the azimuth and elevation DOA joint estimation is usually narrower and sharper, concentrated in the region closer to the true values. In this case, the estimation results are more reliable and accurate, as shown in Figure 3b. According to the above analysis, we present Conclusion 1.

**Conclusion** **1.** *In the high-SNR region, the joint a posteriori PDF* $p\left(\vartheta ,\theta \left|z\right.\right)$ *follows a bivariate Gaussian distribution. Let $v={\left[\begin{array}{cc} \vartheta & \theta \end{array}\right]}^{T},k={\left[\begin{array}{cc} \vartheta_{0} & \theta_{0} \end{array}\right]}^{T}$; then, $p\left(\vartheta ,\theta \left|z\right.\right)$ can be rewritten as*

(22) $$p\left(\vartheta ,\theta \left|z\right.\right)\approx \frac{1}{2\pi \sqrt{\left|C_{\vartheta ,\theta }\right|}}\exp\left\{\frac{-{\left(v-k\right)}^{Τ}C_{\vartheta ,\theta }^{-1}\left(v-k\right)}{2}\right\},$$

*where*

(23) $$C_{\vartheta ,\theta }=\left[\begin{array}{cc} {\left(2Q\rho^{2}L^{2}\sin^{2}\theta_{0}\right)}^{-1} & 0\\ 0 & {\left(2Q\rho^{2}L^{2}\cos^{2}\vartheta_{0}\right)}^{-1} \end{array}\right]$$

*is the covariance matrix of azimuth and elevation DOAs, and* $L^{2}=\pi^{2}\left(Q+1\right)\left(Q-1\right)d^{2}/\left(3\lambda^{2}\right)$ *represents normalization aperture width.*

**Proof.**  See Appendix A. □

Based on the results presented in the covariance matrix in (23), further analysis reveals that in the high-SNR condition, azimuth and elevation DOAs are independent of each other. Thus, marginal distributions of azimuth and elevation DOAs can be obtained, respectively, as

(24) $$\begin{array}{ll} p\left(\vartheta \left|z\right.\right) & \approx \eta \exp\left(-\frac{1}{2}2Q\rho^{2}L^{2}\sin^{2}\theta_{0}{\left(\vartheta -\vartheta_{0}\right)}^{2}\right)\\ & =\frac{1}{\sqrt{2\pi \sigma_{\vartheta }^{2}}}\exp\left(-\frac{{\left(\vartheta -\vartheta_{0}\right)}^{2}}{2\sigma_{\vartheta }^{2}}\right), \end{array}$$

where $\sigma_{\vartheta }^{2}={\left(2Q\rho^{2}L^{2}\sin^{2}\theta_{0}\right)}^{-1}$.

(25) $$\begin{array}{ll} p\left(\theta \left|z\right.\right) & \approx \eta \exp\left(-\frac{1}{2}2Q\rho^{2}L^{2}\cos^{2}\theta_{0}{\left(\theta -\theta_{0}\right)}^{2}\right)\\ & =\frac{1}{\sqrt{2\pi \sigma_{\theta }^{2}}}\exp\left(-\frac{{\left(\theta -\theta_{0}\right)}^{2}}{2\sigma_{\theta }^{2}}\right), \end{array}$$

where $\sigma_{\theta }^{2}={\left(2Q\rho^{2}L^{2}\cos^{2}\vartheta_{0}\right)}^{-1}$.

In conclusion, the *a posteriori* PDF of the azimuth and elevation DOAs follows independent 1D Gaussian distributions in the high-SNR region.

## 4. Joint Entropy Error Bound

**Figure 4:**
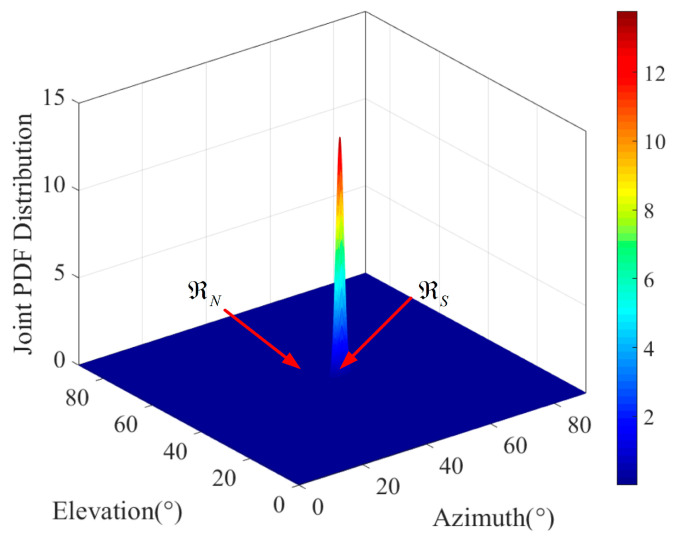
The signal region and noise region.

The variation in the shape of the *a posteriori* distribution, as observed in Figure 4, represents the accuracy of parameter estimation. When the parameter estimation accuracy is high, the *a posteriori* distribution typically exhibits a concentrated, peaked, and compact shape. Conversely, when the parameter estimation accuracy is low, the *a posteriori* distribution may display a dispersed, blurred, or multi-modal shape. *A posteriori* entropy is a crucial concept in Bayesian statistics, used to quantify the uncertainty of parameter estimation under the known received signal. The *a posteriori* entropy can be calculated as

(26) $$h\left(\vartheta ,\theta \left|z\right.\right)=-E_{z}\left[\int \int p\left(\vartheta ,\theta \left|z\right.\right)\log_{2}p\left(\vartheta ,\theta \left|z\right.\right)d\vartheta d\theta \right].$$

Then, the EEB for azimuth and elevation DOAs is provided in Conclusion 2.

**Conclusion** **2.** *The EEB for azimuth and elevation DOAs is defined as the entropy power of azimuth and elevation DOAs, which is calculated as*

(27) $$\sigma_{\mathrm{AEEEB}}^{2}\left(\vartheta ,\theta \left|z\right.\right)=\frac{2^{2h\left(\vartheta ,\theta \left|z\right.\right)}}{{\left(2\pi e\right)}^{2}}.$$

For the sake of convenience, $\sigma_{\mathrm{AEEEB}}^{2}\left(\vartheta ,\theta \left|z\right.\right)$ is written as $\sigma_{\mathrm{AEEEB}}^{2}$ hereafter.

It is apparent that the AEEEB solely depends on the received signal, the system noise, and the SNR. Consequently, the AEEEB remains unaffected by any particular estimator and serves as a universal measure of the performance bound for L-ULA. A smaller EEB indicates a more concentrated *a posteriori* distribution of the parameter, indicating higher accuracy in parameter estimation.

### 4.1. The Joint Asymptotic Lower Bound

According to Conclusion 1, we know that $p\left(\vartheta ,\theta \left|z\right.\right)$ follows a 2D Gaussian distribution in the high-SNR region. Thus, the *a posteriori* entropy is written as

(28) $$\begin{array}{ll} h\left(\vartheta ,\theta \left|z\right.\right) & =\log_{2}\left(2\pi e{\left|C_{\vartheta ,\theta }\right|}^{1/2}\right)\\ & =\log_{2}\left(\frac{\pi e}{Q\rho^{2}L^{2}\sin\theta_{0}\cos\vartheta_{0}}\right). \end{array}$$

Substituting (28) into (27), we obtain the joint asymptotic lower bound for the AEEEB, provided in Conclusion 3.

**Conclusion** **3.** *The joint asymptotic lower bound for the AEEEB is calculated as*

(29) $$\sigma_{\mathrm{AEEEB}}^{2}=\frac{1}{4Q^{2}\rho^{4}L^{4}\sin^{2}\theta_{0}\cos^{2}\vartheta_{0}}.$$

From (29), it is evident that the asymptotic lower bound is inversely proportional to the number of elements, normalized aperture width, and the square of the SNR. This also demonstrates that the AEEEB remains unaffected by any particular estimator. In addition, due to the independence of azimuth and elevation in the high-SNR region, the AEEEB can also be divided into two parts, written as

(30) $$\sigma_{\mathrm{AEEEB}}^{2}=\sigma_{\mathrm{AEEB}}^{2}\times \sigma_{\mathrm{EEEB}}^{2},$$

where $\sigma_{\mathrm{AEEB}}^{2}$ is the AEEB and $\sigma_{\mathrm{EEEB}}^{2}$ is the EEEB.

The joint asymptotic lower bound for AEEB satisfies

(31) $$\sigma_{\mathrm{AEEB}}^{2}={\left(2Q\rho^{2}L^{2}\sin^{2}\theta_{0}\right)}^{-1},$$

and the joint asymptotic lower bound for EEEB satisfies

(32) $$\sigma_{\mathrm{EEEB}}^{2}={\left(2Q\rho^{2}L^{2}\cos^{2}\vartheta_{0}\right)}^{-1}.$$

### 4.2. The Approximate Global Performance Bound

The joint global performance bounds play a crucial role in parameter estimation, as they provide insights into the accuracy assessment of parameter estimation results and guide parameter tuning. In this part, we derive the approximate performance bound of the AEEEB.

It can be seen in Figure 3 that the *a posteriori* distribution of the azimuth and elevation DOAs has a peak at $\left(\vartheta_{0},\theta_{0}\right)$, and other regions are relatively flat. Therefore, we divide the whole observation region into two subregions. The first region is the signal region ${ℜ}_{S}$ (the signal takes the dominant position), which satisfies $G\left(\vartheta ,\theta \right)\gg 0$, the other region is the noise region ${ℜ}_{N}$, which satisfies $G\left(\vartheta ,\theta \right)\approx 0$, as shown in Figure 4.

Let

(33) $$V_{S}=I_{0}\left(2\rho^{2}\left|G\left(\vartheta ,\theta \right)\;+\;\frac{1}{\alpha }F\left(\vartheta ,\theta ,w\right)\right|\right),$$

(34) $$V_{N}=I_{0}\left(\frac{2\rho^{2}}{\alpha }\left|F\left(\vartheta ,\theta ,w\right)\right|\right).$$

$p\left(\vartheta ,\theta \left|z\right.\right)$ is rewritten as

(35) $$p\left(\vartheta ,\theta \left|z\right.\right)\approx \frac{V_{s}}{R_{s}+R_{N}},$$

where

(36) $$R_{S}=\int \int_{R_{S}}V_{s}d\vartheta d\theta ,$$

(37) $$R_{N}=\int \int_{R_{N}}V_{N}d\vartheta d\theta .$$

Substituting (37) into (33), we obtain the *a posteriori* entropy, written as

(38) $$\begin{array}{ll} h\left(\vartheta ,\theta \left|z\right.\right) & =-\iint \frac{V_{S}}{R_{S}+R_{N}}\log\frac{V_{S}}{R_{S}+R_{N}}d\vartheta d\theta \\ & =-\iint_{R_{S}}\frac{V_{S}}{R_{S}+R_{N}}\log\frac{V_{S}}{R_{S}+R_{N}}d\vartheta d\theta -\iint_{R_{N}}\frac{V_{S}}{R_{S}+R_{N}}\log\frac{V_{S}}{R_{S}+R_{N}}d\vartheta d\theta . \end{array}$$

The first term in (37) is further written as

(39) $$\begin{array}{l} -\iint_{R_{S}}\frac{V_{S}}{R_{S}+R_{N}}\log\frac{V_{S}}{R_{S}+R_{N}}d\vartheta d\theta \\ =-\iint_{R_{S}}\frac{V_{S}}{R_{S}+R_{N}}\log\frac{V_{S}}{R_{S}}d\vartheta d\theta -\iint_{R_{S}}\frac{V_{S}}{R_{S}+R_{N}}\log\frac{R_{S}}{R_{S}+R_{N}}d\vartheta d\theta \\ =\frac{R_{S}}{R_{S}+R_{N}}\left(-\iint_{R_{S}}\frac{V_{S}}{R_{S}}\log\frac{V_{S}}{R_{S}}d\vartheta d\theta \right)-\frac{R_{S}}{R_{S}+R_{N}}\log\frac{R_{S}}{R_{S}+R_{N}}\\ \approx p_{s}H_{s}-p_{s}\log p_{s}, \end{array}$$

where $H_{s}$ represents the *a posteriori* entropy in the signal region, and $p_{s}$ represents the proportion of the *a posteriori* entropy of the signal region. In addition, it is noted that the decomposition of terms is neglected, i.e., $\frac{V_{S}}{R_{S}+R_{N}}=\frac{V_{S}}{R_{S}}\cdot \frac{R_{S}}{R_{S}+R_{N}}$ and $R_{S}+R_{N}\approx R_{S}$.

The second term in (38) is further written as

(40) $$\begin{array}{l} -\iint_{R_{N}}\frac{V_{N}}{R_{S}+R_{N}}\log\frac{V_{N}}{R_{S}+R_{N}}d\vartheta d\theta \\ =-\iint_{R_{N}}\frac{V_{N}}{R_{S}+R_{N}}\log\frac{V_{N}}{R_{N}}d\vartheta d\theta -\iint_{R_{N}}\frac{V_{N}}{R_{S}+R_{N}}\log\frac{R_{N}}{R_{S}+R_{N}}d\vartheta d\theta \\ =\frac{R_{N}}{R_{S}+R_{N}}\left(-\iint_{R_{N}}\frac{V_{N}}{R_{N}}\log\frac{V_{N}}{R_{N}}d\vartheta d\theta \right)-\frac{V_{N}}{R_{S}+R_{N}}\log\frac{V_{N}}{R_{S}+R_{N}}\\ \approx \left(1-p_{s}\right)H_{n}-\left(1-p_{s}\right)\log\left(1-p_{s}\right), \end{array}$$

where $H_{n}$ represents the *a posteriori* entropy in the signal region, and $\left(1-p_{s}\right)$ represents the proportion of the *a posteriori* entropy of the noise region. In addition, it is noted that the decomposition of terms is neglected, i.e., $\frac{V_{N}}{R_{S}+R_{N}}=\frac{V_{N}}{R_{S}}\cdot \frac{R_{S}}{R_{S}+R_{N}}$ and $R_{S}+R_{N}\approx R_{N}$.

Eventually, we can obtain the a posteriori entropy in the whole region, which is approximately written as

(41) $$h\left(\vartheta ,\theta \left|z\right.\right)=p_{s}H_{s}+\left(1-p_{s}\right)H_{n}+H\left(p_{s}\right),$$

where $H\left(p_{s}\right)$ denotes the probability of $\left(\vartheta ,\theta \right)$ occurring at $\left(\vartheta_{0},\theta_{0}\right)$, and it is expressed as

(42) $$H\left(p_{s}\right)=-p_{s}\log p_{s}-\left(1-p_{s}\right)\log\left(1-p_{s}\right).$$

In Appendix B, the $R_{N}$ in the low-SNR region is derived, which is written as

(43) $$\begin{array}{ll} R_{N} & =\int \int_{R_{N}}V_{N}d\vartheta d\theta \\ & =\left|\Theta \right|\left|\Xi \right|\int_{0}^{\infty }\eta I_{0}\left(\left|h_{n}\left(\vartheta ,\theta \right)\right|\right)f\left(\left|h_{n}\left(\vartheta ,\theta \right)\right|\right)d\left|h_{n}\left(\vartheta ,\theta \right)\right|\\ & \approx \left|\Theta \right|\left|\Xi \right|\exp\left(2Q\rho^{2}\right). \end{array}$$

In Appendix C, the $H_{n}$ in the low-SNR region is derived, which is shown as

(44) $$\begin{array}{ll} H_{n} & =-\left|\Theta \right|\left|\Xi \right|\left[\eta \ln\left(\frac{\eta }{\sqrt{2\pi }}\right)\exp\left(2Q\rho^{2}\right)\right.\left.+\sqrt{2\pi Q}\eta \rho \exp\left(Q\rho^{2}\right)-\eta \exp\left(2Q\rho^{2}\right)\ln\left(\sqrt{4Q\rho^{2}}\right)\right]\\ & \approx \ln\left(\frac{2\sqrt{2\pi Q\rho^{2}}\left|\Theta \right|\left|\Xi \right|}{\exp\left(\sqrt{2\pi Q\rho^{2}}-2Q\rho^{2}\right)}\right). \end{array}$$

In Appendix D, the $R_{S}$ in the high-SNR region is derived, which is written as

(45) $$R_{S}\approx \frac{\sqrt{\pi }\exp\left(4Q\rho^{2}+1\right)}{\sqrt{2\left(4Q\rho^{2}+1\right)}\left(Q^{3}-Q\right)\rho^{2}L^{2}\sin\theta_{0}\cos\vartheta_{0}}.$$

And $H_{S}$ is calculated in (28).

Based on (43) and (45), $p_{s}$ is calculated as

(46) $$\begin{array}{ll} p_{s} & =\frac{R_{S}}{R_{S}+R_{N}}\\ & =\frac{\sqrt{\pi }\exp\left(2Q\rho^{2}+1\right)}{\sqrt{\pi }\exp\left(2Q\rho^{2}+1\right)+\left|\Theta \right|\left|\Xi \right|\sqrt{2\left(4Q\rho^{2}+1\right)}Q\rho^{2}L^{2}\sin\theta_{0}\cos\vartheta_{0}}. \end{array}$$

**Figure 5:**
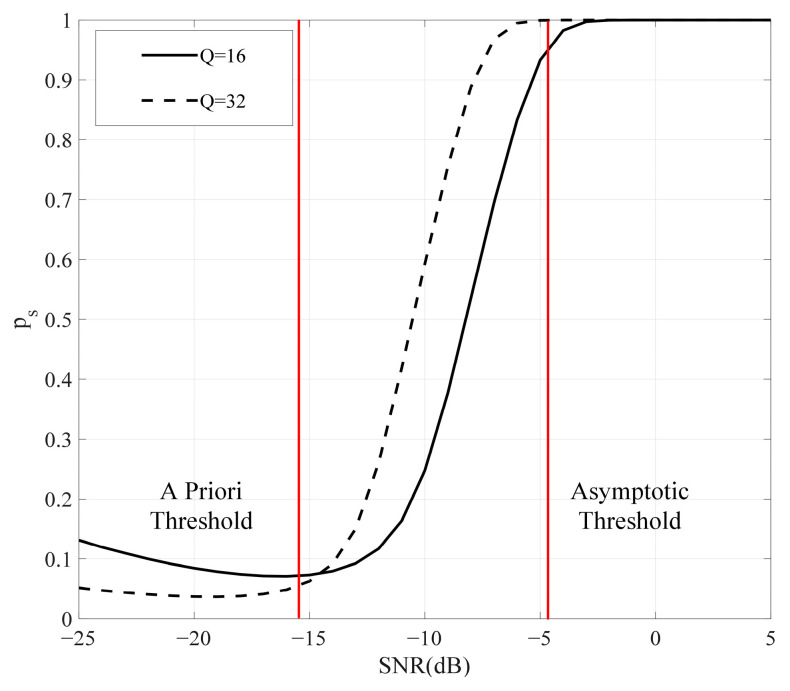
The relationship between $p_{s}$ and SNR under different number of elements.

According to (46), the relationship between $p_{s}$ and the SNR is illustrated in Figure 5. The black lines represent the values of $p_{s}$ under different element conditions, while the red lines indicate the threshold. $p_{s}$ divides the SNR range into three regions. The first region is the prior region, where $p_{s}\approx 0$ and the threshold is referred to as the *a priori* threshold. The second region is the asymptotic region, where $p_{s}\approx 1$ and the threshold is known as the asymptotic threshold. The region between these two thresholds is the medium SNR. However, it can be clearly observed that $p_{s}$ increases significantly in the low-SNR region, which can be attributed to the approximate bias of $R_{S}$ in the low-SNR region. However, as the number of elements increases, the error of $p_{s}$ decreases in the *a priori* region.

Substituting (28) and (44) into (41), we obtain the joint expression for a posteriori entropy, given as

(47) $$h\left(\vartheta ,\theta \left|z\right.\right)=p_{s}\ln\left(\frac{\pi e}{Q\rho^{2}L^{2}\sin\theta_{0}\cos\vartheta_{0}}\right)+\left(1-p_{s}\right)\ln\left(\frac{2\sqrt{2\pi Q\rho^{2}}\left|\Theta \right|\left|\Xi \right|}{\exp\left(\sqrt{2\pi Q\rho^{2}}-2Q\rho^{2}\right)}\right)+H\left(p_{s}\right).$$

Finally, substituting (47) and (26), we calculate the joint performance expression for AEEEB in Conclusion 4.

**Conclusion** **4.** *The approximate performance expression for the AEEEB is expressed as*

(48) $$\begin{array}{ll} \sigma_{\mathrm{AEEEB}}^{2}\left(\vartheta ,\theta \left|z\right.\right) & =\frac{1}{{\left(2\pi e\right)}^{2}}{\left(\frac{\pi e}{Q\rho^{2}L^{2}\sin\theta_{0}\cos\vartheta_{0}}\right)}^{2p_{s}}\\ & \times {\left(\frac{2\sqrt{2\pi Q\rho^{2}}\left|\Theta \right|\left|\Xi \right|}{\exp\left(\sqrt{2\pi Q\rho^{2}}-2Q\rho^{2}\right)}\right)}^{2-2p_{s}}\times {\left(\frac{1}{p_{s}}\right)}^{2p_{s}}\times {\left(\frac{1}{1-p_{s}}\right)}^{2-2p_{s}}. \end{array}$$

*Upon careful observation of (46) and (48), it can be noticed that the AEEEB is only dependent on the size of the observation interval, the number of elements, and the SNR.*

### 4.3. The Approximate AEEEB for Special Scenarios

The approximate expression in (48) can be used to analyze the performance of the L-shaped array system in special scenarios. In the *a priori* region (the low-SNR region), $p_{s}\approx 0$, and the AEEEB can be rewritten as

(49) $$\sigma_{\mathrm{AEEEB}}^{2}=\frac{1}{{\left(2\pi e\right)}^{2}}\times {\left(\frac{2\sqrt{2\pi Q\rho^{2}}\left|\Theta \right|\left|\Xi \right|}{\exp\left(\sqrt{2\pi Q\rho^{2}}-2Q\rho^{2}\right)}\right)}^{2}.$$

Upon careful observation of (49), we find that the number of elements does not provide a significant gain to the L-shaped array system when the SNR is very low. The main factor that affects performance is the observation interval. Therefore, when the observation interval is fixed, the performance curve of the proposed bound remains almost unchanged in the *a priori* region.

In the asymptotic region (the high-SNR region), $p_{s}\approx 1$, and the AEEEB can be rewritten as

(50) $$\sigma_{\mathrm{AEEEB}}^{2}=\frac{1}{4Q^{2}\rho^{4}L^{4}\sin^{2}\theta_{0}\cos^{2}\vartheta_{0}}.$$

Upon careful observation of (50), we find that the observation interval does not affect the performance of the L-shaped array system, which is the same result as that deduced in [17] (see (26)–(28)). The main factors that affect performance are the number of elements and the SNR. The AEEEB is inversely proportional to the fourth power of the SNR and the square of the number of array elements.

### 4.4. Comparison with Cramér–Rao Bound

The CRB establishes a lower bound on the variance of any unbiased estimator [31]. Here, we provide the CRB for azimuth and elevation DOA joint estimation as a benchmark to which to compare the proposed AEEEB.

The Fisher information matrix (FIM) for azimuth and elevation DOAs is expressed as

(51) F=2ρ2Re∂Aϑ,θ∂ϑH∂Aϑ,θ∂ϑ∂Aϑ,θ∂ϑH∂Aϑ,θ∂θ∂Aϑ,θ∂θH∂Aϑ,θ∂ϑ∂Aϑ,θ∂θH∂Aϑ,θ∂θ.

Using (51), we construct the FIM; we have

(52) $$F_{\vartheta ,\theta }=\left[\begin{array}{cc} F_{\vartheta ,\vartheta } & F_{\vartheta ,\theta }\\ F_{\theta ,\vartheta } & F_{\theta ,\theta } \end{array}\right].$$

The inverse of the FIM is the CRB matrix, which is written as

(53) $$\left[\begin{array}{cc} {\mathrm{CRB}}_{\vartheta ,\vartheta } & *\\ {}* & {\mathrm{CRB}}_{\theta ,\theta } \end{array}\right]={\left[\begin{array}{cc} F_{\vartheta ,\vartheta } & F_{\vartheta ,\theta }\\ F_{\theta ,\vartheta } & F_{\theta ,\theta } \end{array}\right]}^{-1}$$

where * refers to the terms that do not contribute to the CRB.

The ${\mathrm{CRB}}_{\vartheta ,\vartheta }$ is calculated as

(54) $${\mathrm{CRB}}_{\vartheta ,\vartheta }=\frac{F_{\theta ,\theta }}{F_{\vartheta ,\vartheta }F_{\theta ,\theta }-{F_{\vartheta ,\theta }}^{2}}={\left(2Q\rho^{2}L^{2}\sin^{2}\theta \right)}^{-1}.$$

The ${\mathrm{CRB}}_{\theta ,\theta }$ is calculated as

(55) $${\mathrm{CRB}}_{\theta ,\theta }=\frac{F_{\vartheta ,\vartheta }}{F_{\vartheta ,\vartheta }F_{\theta ,\theta }-{F_{\vartheta ,\theta }}^{2}}={\left(2Q\rho^{2}L^{2}\cos^{2}\vartheta \right)}^{-1}.$$

The CRB for azimuth and elevation DOA joint estimation is written as

(56) $$\begin{array}{ll} \mathrm{CRB} & ={\mathrm{CRB}}_{\vartheta ,\vartheta }\cdot {\mathrm{CRB}}_{\theta ,\theta }\\ & ={\left(4Q^{2}\rho^{4}L^{4}\sin^{2}\theta \cos^{2}\vartheta \right)}^{-1}. \end{array}$$

Based on (50) and (56), we find that the performance lower bound of the AEEEB is consistent with the CRB.

**Proof.**  See Appendix E. □

### 4.5. Comparison with Mean Squared Error

The MSE is calculated as

(57) $$\begin{array}{l} \mathrm{MSE}\left(\hat{x}\right)=E{\left(\hat{x}-x\right)}^{2}\\ =D\left(\hat{x}-x\right)+{\left[E\left(\hat{x}-x\right)\right]}^{2}\\ =D\left(\hat{x}\right)+{\left[E\left(\hat{x}\right)-x\right]}^{2}\\ =\sigma^{2}. \end{array}$$

For the unbiased estimator, the second term in (57) is zero. Thus, $\mathrm{MSE}\left(\hat{x}\right)=\sigma^{2}$ [32].

We further analyze the relationship between the MSE and the EEB. In the low-SNR region, assuming that the *a priori* distribution of the parameters follows a uniform distribution, the differential entropy $h\left(x\right)=\log_{2}\left(b-a\right)$, where $x\in \left[a,b\right]$. Substituting $h\left(x\right)$ into the calculation formula of the EEB, we calculate the EEB as $\sigma_{\mathrm{EE}}^{2}={\left(b-a\right)}^{2}/\left(2\pi e\right)$, and the variance is calculated as $\sigma^{2}={\left(b-a\right)}^{2}/12$. For azimuth and elevation DOA joint estimation, the AEEEB is calculated as $\sigma_{\mathrm{AEEEB}}^{2}={\left|\Theta \right|}^{2}{\left|\Xi \right|}^{2}/{\left(2\pi e\right)}^{2}$, and the variance is calculated as ${\left|\Theta \right|}^{2}{\left|\Xi \right|}^{2}/144$. Therefore, the MSE is slightly larger than the AEEEB under the low-SNR condition.

In the high-SNR region, the parameters follow a Gaussian distribution under the assumption of a Gaussian channel. The differential entropy $h\left(x\right)=1/2\log_{2}\left(2\pi e\sigma^{2}\right)$, where $\sigma^{2}$ denotes the variance. Substituting $h\left(x\right)$ into the calculation formula of the EEB, we calculate the EEB as $\sigma^{2}$; i.e., the EEB equals the variance. For azimuth and elevation DOA joint estimation, the AEEEB is calculated as $\det\left(C_{\vartheta ,\theta }\right)$. The AEEEB is consistent with the product of the ${\mathrm{CRB}}_{\vartheta ,\vartheta }$ and the ${\mathrm{CRB}}_{\theta ,\theta }$. Therefore, the AEEEB, the MSE, and the CRB are consistent with each other. When $\det\left(C_{\vartheta ,\theta }\right)$ is not a diagonal matrix, the AEEEB may be smaller than the product of the CRB. This occurs as a result of the association between azimuth and elevation DOAs, leading to diminished uncertainty.

## 5. Simulation Testing

In Section 5, a comprehensive set of numerical simulations was performed to rigorously evaluate our theoretical derivations for azimuth and elevation DOA joint estimation. We chose the MATLAB R2016a platform for our versatile simulation testing needs. The simulation parameters are detailed in Table 2.

**Figure 6:**
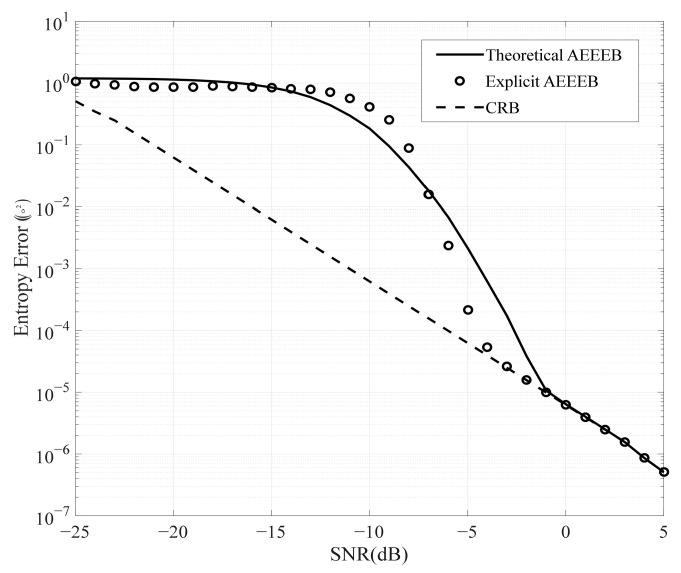
Comparison of the performance curves for the theoretical AEEEB with the approximate AEEEB.

The first study compares the performance curves of the theoretical AEEEB with the approximate AEEEB. The theoretical AEEEB is calculated by (18), (26), and (27), the approximate AEEEB is calculated by (48), and the CRB is obtained by (56). The simulation results are plotted in Figure 6. In the low-SNR region, the approximate AEEEB curve is slightly below the theoretical AEEEB curve, which is attributed to the increase in $p_{s}$ (see Figure 5). In the medium-SNR region, the difference falls within an acceptable range. In the high-SNR region, the approximate AEEEB curve and the theoretical AEEEB gradually approach the CRB curves. Therefore, the approximate AEEEB can replace the theoretical AEEEB in the whole SNR region.

**Figure 7:**
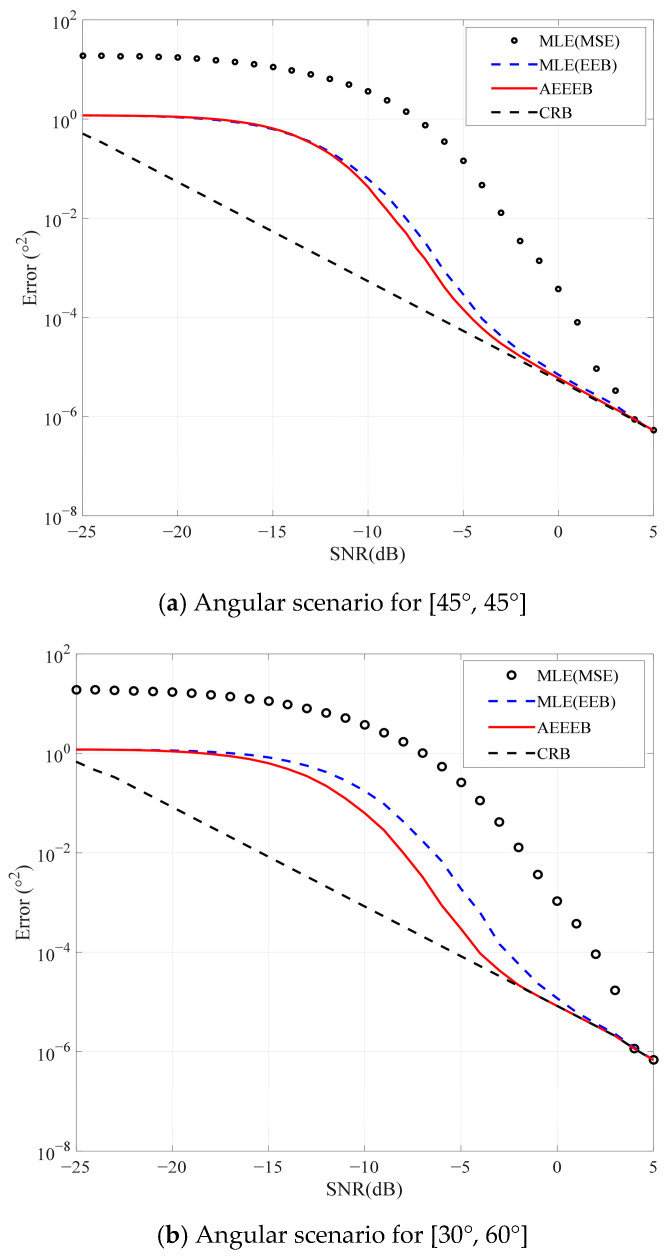
Comparison of the different performance bounds.

We then plot various performance bounds for comparison with the AEEEB, as shown in Figure 7. The MLE is executed by conducting a 2D search to identify the PDF’s peak value in (14), as shown using a black line marked with circles. The calculation formula of the MSE for 2D DOA can be expressed as $\mathrm{MSE}=E_{\theta }\left[{\left(\theta -\theta_{0}\right)}^{2}\right]+E_{\vartheta }\left[{\left(\vartheta -\vartheta_{0}\right)}^{2}\right]$. The AEEEB is calculated by (27), and the CRB is calculated by (56), displayed using a red line and a black dashed line, respectively. In the asymptotic region, the AEEEB gradually approaches the CRB, as the AEEEB utilizes the entropy of Gaussian distribution while the CRB utilizes the FIM (see (50) and (56)). Outside the asymptotic region, the AEEEB exhibits a tighter performance bound compared to the CRB. In addition, we also provide the AEEEB on the predictive capability of the empirical performance of the MLE estimators. The EEB of the MLE is calculated based on the empirical a posteriori PDF, which is obtained through a statistical search of parameter estimation results. It can be seen that the AEEEB accurately predicts the empirical performance of MLE estimators in the asymptotic region. Outside the asymptotic region, the AEEEB is tighter than the CRB. This is consistent with the results of 1D DOA estimation [26]. In addition, to validate the generality of the proposed AEEEEB, we have added a set of angular scenarios. It is observed that the performance of the proposed AEEEEB is consistent in different angular scenarios. Outside the asymptotic region, the AEEEEB exhibits tighter performance compared to the CRB, while within the asymptotic region, the AEEEEB degrades to the CRB.

**Figure 8:**
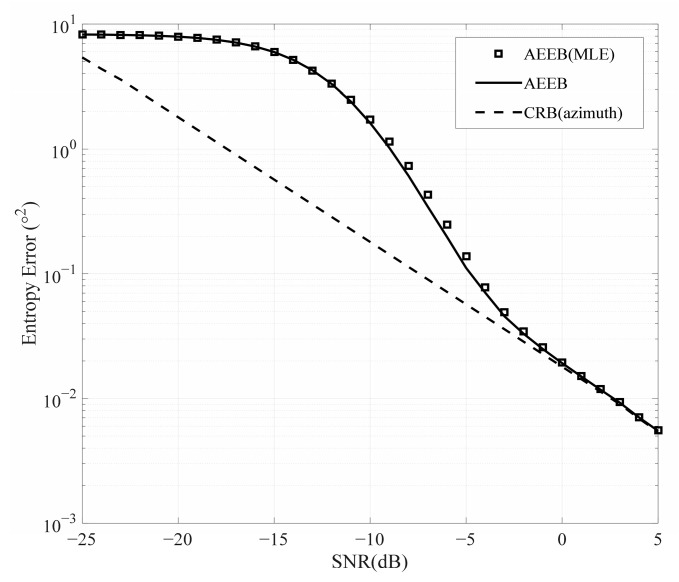
Comparison of the different performance bounds (azimuth estimation).

In addition, there is a special scenario where we only need to estimate the azimuth or elevation. Therefore, we further validate the AEEEB for azimuth estimation, as shown in Figure 8. The MLE is executed by conducting a 1D search to identify the PDF’s peak value in (24), the AEEB is calculated by (31), and the CRB is calculated by (54). It can be observed that the AEEB and the CRB converge asymptotically in the high-SNR region, validating the derivation presented in Section 4.4. Outside the high-SNR region, the AEEB provides a better prediction of the performance of the MLE compared to the CRB.

**Figure 9:**
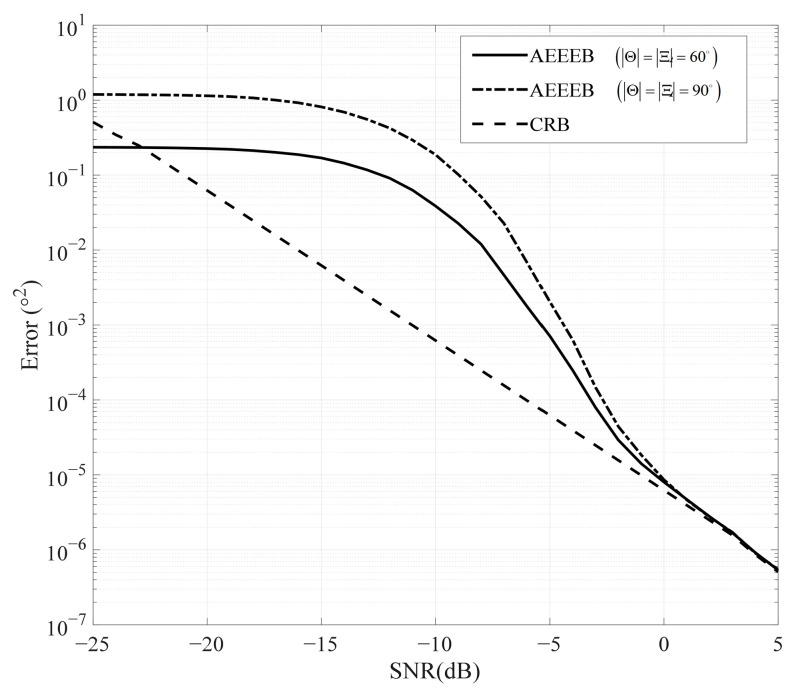
Comparison of AEEEBs with different observation intervals.

The final study examines the relationship between the observation interval and the AEEEB, as shown in Figure 9. In the low-SNR region, the AEEEB is directly proportional to the square of the observation interval. Then, the differences between the AEEEBs diminish as the SNR increases. In the high-SNR region, the AEEEB gradually aligns with the CRB. In addition, we can clearly see that the smaller observation intervals lead to a faster convergence of the AEEEB. Thus, we can conclude that the observation interval only affects the performance of the AEEEB in the low-SNR region, which aligns with the derivation in Section 4.3.

## 6. Conclusions

In this paper, we propose an AEEEB for evaluating azimuth and elevation DOA joint estimation in an L-shaped array. Unlike 1D DOA estimation, 2D DOA estimation requires considering the matching process between the estimated and true values. Based on a general signal model, we derived the joint a posteriori PDF for azimuth and elevation DOAs and then obtained the theoretical AEEEB using the a posteriori entropy. In addition to the theoretical AEEEB, we also derived an approximate AEEEB expression. The expression is not only simple in form but also highlights the impact of array system parameters on estimation accuracy. Simulation results demonstrate that the approximate AEEEB aligns well with the theoretical AEEEB. Furthermore, we compared the AEEEB with the CRB, and the results indicated that the AEEEB provided a globally efficient bound. Additionally, the derived approximate AEEEB is comprehensive as it captures the impact of the observation interval, the number of arrays, and the SNR on the performance bound. Finally, by introducing the AEEEB, researchers can evaluate different DOA algorithms under a unified standard, reducing inconsistencies in results due to different evaluation methods. This is crucial for the reproducibility of academic research and engineering applications. While the AEEEB itself does not directly enhance existing algorithms, it can reveal the limitations of current algorithms in various scenarios, providing direction for future algorithm improvements. For instance, researchers can optimize algorithms that perform poorly in AEEEB evaluations. In future work, we will investigate the joint estimation of azimuth and elevation DOAs for multiple-source situations and explore the angle resolution of multiple sources.

## Figures and Tables

**Table 1:** The list of symbols.

Symbols	Description	Symbols	Description
${\left[\cdot \right]}^{T}$	Transpose	$E\left\{\cdot \right\}$	Statistical expectation
${\left(\cdot \right)}^{*}$	Conjugate transpose	${\left\|\cdot \right\|}_{2}$	Euclidean norm
$\det\left(\cdot \right)$	Determinant of a matrix	⊙	Hadamard product
Re·	Real part	$\left(\hat{\cdot }\right)$	Estimation value for parameter
Im·	Imaginary part	$D\left(\hat{\cdot }\right)$	Variance

**Table 2:** The basic parameter settings.

Parameter	Value	Parameter	Value
SNR region	[−25 dB, 5 dB]	Angle range	[0°, 90°]
Array element interval	*d* = 1 m	Step size in search	0.1°
Number of array elements	Q = 16	Actual location	[45°, 45°] [30°, 60°]
Wavelength	λ=2m	Number of Monte Carlo experiments	1000
Reflection coefficient	$\alpha_{0}=1$	Source propagation velocity	3 × 10^8^ m/s

**Figure A1:**
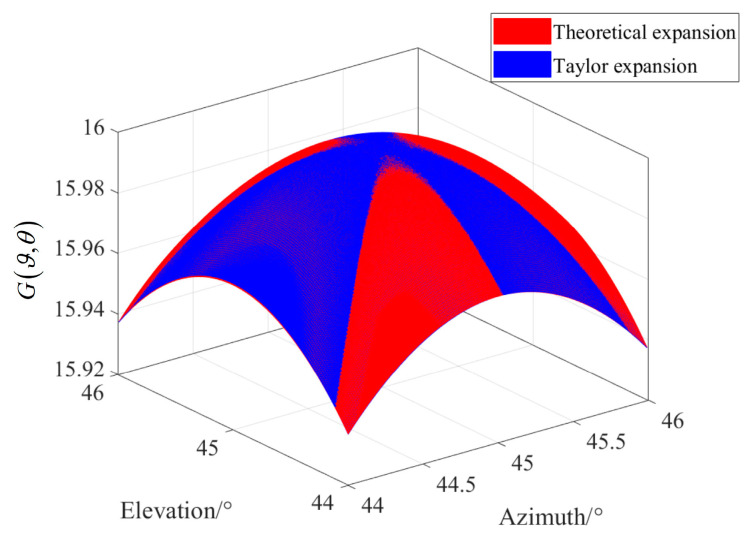
The size of $G\left(\vartheta ,\theta \right)$. *Q* = 8, *d* = 1, $\left(\vartheta_{0},\theta_{0}\right)=\left(45^{{}^{\circ}},45^{{}^{\circ}}\right)$.

**Figure A2:**
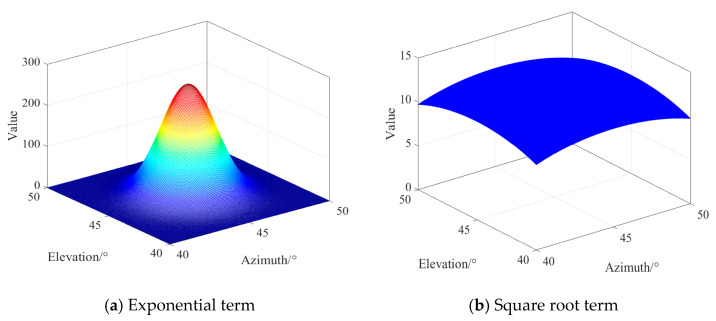
The exponential term and the square root term in the observation region. *Q* = 8, *d* = 1, SNR = 0 dB.

## Data Availability

The data presented in this study are available on request from the corresponding author. The data are not publicly available due to the confidentiality policy of our research group.

## Appendix A

In the high-SNR region, it can be seen that the influence of noise on the signal is masked, which does not significantly impact the quality of the received signal. In this case, the influence of noise in (18) can be ignored. Therefore, (18) can be approximated as

(A1) $$p\left(\vartheta ,\theta \left|z\right.\right)\approx \frac{I_{0}\left(2\rho^{2}\left|G\left(\vartheta ,\theta \right)\right|\right)}{\int_{0}^{\left|\Xi \right|}\int_{0}^{\left|\Theta \right|}I_{0}\left(2\rho^{2}\left|G\left(\vartheta ,\theta \right)\right|\right)d\vartheta d\theta }.$$

To obtain the simple form of (22), the Taylor expansion of $G\left(\vartheta ,\theta \right)$ at $\left(\vartheta_{0},\theta_{0}\right)$ yields is used, nearly equivalent to

(A2) $$\begin{array}{l} \left|G\left(\vartheta ,\theta \right)\right|\approx \left|G_{x}\left(\vartheta ,\theta \right)+G_{y}\left(\vartheta ,\theta \right)\right|\\ =2Q\\ \;+\left[\frac{1}{2}\frac{\pi^{2}d^{2}}{3\lambda^{2}}\left(\left(Q-Q^{3}\right)\sin^{2}\vartheta_{0}\sin^{2}\theta_{0}+\left(Q-Q^{3}\right)\sin^{2}\vartheta_{0}\cot^{2}\vartheta_{0}\sin^{2}\theta_{0}\right)\right]{\left(\vartheta -\vartheta_{0}\right)}^{2}\\ \;+\left[\frac{\pi^{2}d^{2}}{3\lambda^{2}}\left(\left(Q-P^{3}\right)-\left(Q-Q^{3}\right)\right)\cos\vartheta_{0}\cos\theta_{0}\sin\vartheta_{0}\sin\theta_{0}\right]\left(\vartheta -\vartheta_{0}\right)\left(\theta -\theta_{0}\right)\\ \;+\left[\frac{1}{2}\frac{\pi^{2}d^{2}}{3\lambda^{2}}\left(\left(Q-Q^{3}\right)\cos^{2}\vartheta_{0}\cos^{2}\theta_{0}+\left(Q-Q^{3}\right)\cos^{2}\vartheta_{0}\sin^{2}\theta_{0}\right)\right]{\left(\theta -\theta_{0}\right)}^{2} \end{array}$$

where

(A3) $$\begin{array}{ll} G_{x}\left(\vartheta ,\theta \right) & =Q+\left[\frac{1}{2}\frac{\pi^{2}d^{2}}{3\lambda^{2}}\left(Q-Q^{3}\right)\sin^{2}\vartheta_{0}\sin^{2}\theta_{0}\right]{\left(\vartheta -\vartheta_{0}\right)}^{2}\\ & +\left[-\frac{\pi^{2}d^{2}}{3\lambda^{2}}\left(Q-Q^{3}\right)\cos\vartheta_{0}\cos\theta_{0}\sin\vartheta_{0}\sin\theta_{0}\right]\left(\vartheta -\vartheta_{0}\right)\left(\theta -\theta_{0}\right)\\ & +\left[\frac{1}{2}\frac{\pi^{2}d^{2}}{3\lambda^{2}}\left(Q-Q^{3}\right)\cos^{2}\vartheta_{0}\cos^{2}\theta_{0}\right]{\left(\theta -\theta_{0}\right)}^{2}, \end{array}$$

(A4) $$\begin{array}{ll} G_{y}\left(\vartheta ,\theta \right) & =Q+\left[\frac{1}{2}\frac{\pi^{2}d^{2}}{3\lambda^{2}}\left(Q-Q^{3}\right)\sin^{2}\vartheta_{0}\cot^{2}\vartheta_{0}\sin^{2}\theta_{0}\right]{\left(\vartheta -\vartheta_{0}\right)}^{2}\\ & +\left[\frac{\pi^{2}d^{2}}{3\lambda^{2}}\left(Q-Q^{3}\right)\cos\vartheta_{0}\cos\theta_{0}\sin\vartheta_{0}\sin\theta_{0}\right]\left(\vartheta -\vartheta_{0}\right)\left(\theta -\theta_{0}\right)\\ & +\left[\frac{1}{2}\frac{\pi^{2}d^{2}}{3\lambda^{2}}\left(Q-Q^{3}\right)\sin^{2}\vartheta_{0}\cos^{2}\theta_{0}\right]{\left(\theta -\theta_{0}\right)}^{2}. \end{array}$$

The higher-order terms of the above numerical results can be neglected due to the peak of the *a posteriori* probability distribution at $\left(\vartheta_{0},\theta_{0}\right)$ under high-SNR conditions. The numerical results of the aforementioned Taylor expansion have been verified through Mathematica.

Furthermore, the approximation expansion of $I_{0}\left(x\right)$ is given by

(A5) $$I_{0}\left(x\right)\approx \frac{\exp\left(x\right)}{\sqrt{2\pi x}}\left\{1+\frac{1}{8x}+o\left(\frac{1}{x^{2}}\right)\right\}.$$

Finally, substituting (A2) and (A5) into (A1), we have

(A6) $$\begin{array}{l} p\left(\vartheta ,\theta \left|Z\right.\right)\\ \approx \eta I_{0}\left(2\rho^{2}\left|G\left(\vartheta ,\theta \right)\right|\right)\\ \approx \eta \exp\left[\begin{array}{l} \left[-\frac{1}{2}\left(\rho^{2}L^{2}\right)\left(2\left(Q^{3}-Q\right)\sin^{2}\vartheta_{0}\sin^{2}\theta_{0}+2\left(Q^{3}-Q\right)\sin^{2}\vartheta_{0}\cot^{2}\vartheta_{0}\sin^{2}\theta_{0}\right)\right]{\left(\vartheta -\vartheta_{0}\right)}^{2}\\ +\left[\left(\rho^{2}L^{2}\right)\left(2\left(Q-Q^{3}\right)-2\left(Q-Q^{3}\right)\right)\cos\vartheta_{0}\cos\theta_{0}\sin\vartheta_{0}\sin\theta_{0}\right]\left(\vartheta -\vartheta_{0}\right)\left(\theta -\theta_{0}\right)\\ +\left[-\frac{1}{2}\left(\rho^{2}L^{2}\right)\left(2\left(Q^{3}-Q\right)\cos^{2}\vartheta_{0}\cos^{2}\theta_{0}+2\left(Q-Q^{3}\right)\cos^{2}\theta_{0}\sin^{2}\vartheta_{0}\right)\right]{\left(\theta -\theta_{0}\right)}^{2} \end{array}\right]. \end{array}$$

where $L^{2}=\beta^{2}d^{2}/\lambda^{2}$, $\beta^{2}=\pi^{2}/3$ represents the root mean square bandwidth, and η represents the normalization constant.

Then, we have

(A7) $$\begin{array}{ll} p\left(\vartheta ,\theta \left|z\right.\right) & \approx \eta \exp\left[\begin{array}{l} \left[-\frac{1}{2}\left(\rho^{2}\left(Q^{2}-1\right)L^{2}\right)2Q\sin^{2}\vartheta_{0}\sin^{2}\theta_{0}\left(1+\cot^{2}\vartheta_{0}\right)\right]{\left(\vartheta -\vartheta_{0}\right)}^{2}\\ +\left[-\frac{1}{2}\left(\rho^{2}\left(Q^{2}-1\right)L^{2}\right)2Q\left(\cos^{2}\vartheta_{0}\cos^{2}\theta_{0}+\cos^{2}\vartheta_{0}\sin^{2}\theta_{0}\right)\right]{\left(\theta -\theta_{0}\right)}^{2} \end{array}\right]\\ & =\eta \exp\left[\left[-\frac{1}{2}2Q\rho^{2}L^{2}\sin^{2}\theta_{0}\right]{\left(\vartheta -\vartheta_{0}\right)}^{2}+\left[-\frac{1}{2}2Q\rho^{2}L^{2}\cos^{2}\vartheta_{0}\right]{\left(\theta -\theta_{0}\right)}^{2}\right] \end{array}$$

Let $v={\left[\begin{array}{cc} \vartheta & \theta \end{array}\right]}^{T},k={\left[\begin{array}{cc} \vartheta_{0} & \theta_{0} \end{array}\right]}^{T}$; $p\left(\vartheta ,\theta \left|z\right.\right)$ can be rewritten as

(A8) $$p\left(\vartheta ,\theta \left|z\right.\right)\approx \frac{1}{2\pi \sqrt{\left|C_{\vartheta ,\theta }\right|}}\exp\left\{\frac{-{\left(v-k\right)}^{Τ}C_{\theta ,f}^{-1}\left(v-k\right)}{2}\right\},$$

where

(A9) $$C_{\vartheta ,\theta }=\left[\begin{array}{cc} {\left(2Q\rho^{2}L^{2}\sin^{2}\theta_{0}\right)}^{-1} & 0\\ 0 & {\left(2Q\rho^{2}L^{2}\cos^{2}\vartheta_{0}\right)}^{-1} \end{array}\right]$$

is the covariance matrix of azimuth and elevation DOAs.

According to (A2), the numerical simulations of both theoretical expression and Taylor expansion are plotted to validate the correctness of $G\left(\vartheta ,\theta \right)$, as shown in Figure A1. It is clearly observed that the second-order term of the Taylor expansion provides a good approximation of the theoretical expression. This demonstrates that the Taylor expansion is an effective approximation method in the vicinity region, providing convenience for analysis and computation within that region.

In (A6), the square root term of $I_{0}\left(x\right)$ is neglected. We find that it is not the absolute size of the molecule but rather its flatness that influences the shape of the a posteriori distribution. Figure A2 gives the shapes of both the exponential term and the square root term. It is seen that the exponential term has a much sharper shape compared to the square root term. Therefore, the square root term can be omitted without causing significant approximation errors in the vicinity of the actual value.

## Appendix B

In the low-SNR region, the joint *a posteriori* PDF is written as

(A10) $$p\left(\theta ,\vartheta \left|z\right.\right)=\frac{V_{N}}{R_{N}}=\eta I_{0}\left(\frac{2\alpha }{N_{0}}\left|A^{H}\left(\vartheta ,\theta \right)N\right|\right),$$

where η represents the normalization constant.

Let $\gamma =\frac{2\alpha }{N_{0}}\left|A^{H}\left(\vartheta ,\theta \right)N\right|$; we have

(A11) Reγ2¯=Imγ2¯=4Qρ2.

Consequently, the γ follows the Rayleigh distribution. The PDF is expressed as

(A12) $$f\left(\gamma \right)=\frac{\gamma }{4Q\rho^{2}}\exp\left(-\frac{\gamma^{2}}{8Q\rho^{2}}\right).$$

The expectation of $p\left(\theta ,\vartheta \left|z\right.\right)$ can be expressed as

(A13) $$\begin{array}{ll} E\left(V_{N}\right) & =\int_{0}^{\infty }\eta I_{0}\left(\gamma \right)f\left(\left|\gamma \right|\right)d\gamma \\ & =\int_{0}^{\infty }\eta I_{0}\left(\gamma \right)\frac{\gamma }{4Q\rho^{2}}\exp\left(-\frac{\gamma^{2}}{8Q\rho^{2}}\right)d\gamma \\ & =\sum_{k=0}^{\infty }\eta \frac{2^{2k+1}\Gamma \left(k+1\right){\left(2Q\rho^{2}\right)}^{k+1}}{k!\Gamma \left(k+1\right)2^{2k+1}\left(2Q\rho^{2}\right)}\\ & =\eta \exp\left(2Q\rho^{2}\right). \end{array}$$

It is found that $E\left[p\left(\theta ,\vartheta \left|z\right.\right)\right]$ is a normalization constant. Accordingly, the $p\left(\theta ,\vartheta \left|z\right.\right)$ approximately follows a uniform distribution when the SNR is low. Therefore, we have $\eta \left|\Theta \right|\left|\Xi \right|\exp\left(2Q\rho^{2}\right)\approx 1$; the normalization coefficient is $\eta \approx 1/\left(\left|\Theta \right|\left|\Xi \right|\exp\left(2Q\rho^{2}\right)\right)$.

Finally, $R_{N}$ can be calculated as

(A14) $$\begin{array}{ll} R_{N} & =\int \int_{R_{N}}V_{N}d\vartheta d\theta \\ & =\left|\Theta \right|\left|\Xi \right|\int_{0}^{\infty }I_{0}\left(\gamma \right)f\left(\gamma \right)d\gamma \\ & \approx \left|\Theta \right|\left|\Xi \right|\exp\left(2Q\rho^{2}\right). \end{array}$$

## Appendix C

$H_{n}$ can be calculated as

(A15) $$H_{n}=-\left|\Theta \right|\left|\Xi \right|\int_{0}^{\infty }f\left(\gamma \right)\eta I_{0}\left(\gamma \right)\ln\eta I_{0}\left(\gamma \right)d\gamma .$$

Furthermore, (A15) is approximately written as

(A16) $$H_{n}=-\left|\Theta \right|\left|\Xi \right|\int_{0}^{\infty }f\left(\gamma \right)\eta I_{0}\left(\gamma \right)\ln\eta \frac{\exp\left(\gamma \right)}{\sqrt{2\pi \gamma }}d\gamma .$$

For simplicity, we divide the integral in (A16) into three parts.

I:

(A17) $$\int_{0}^{\infty }f\left(\gamma \right)\eta I_{0}\left(\gamma \right)\ln\frac{\eta }{\sqrt{2\pi }}d\gamma =\int_{0}^{\infty }\frac{\gamma }{4Q\rho^{2}}\exp\left(-\frac{\gamma^{2}}{8Q\rho^{2}}\right)\eta I_{0}\left(\gamma \right)\ln\frac{\eta }{\sqrt{2\pi }}d\gamma ,$$

II:

(A18) $$\int_{0}^{\infty }f\left(\gamma \right)\eta I_{0}\left(\gamma \right)\ln\left(\exp\left(\gamma \right)\right)d\gamma =\int_{0}^{\infty }\frac{\gamma }{4Q\rho^{2}}\exp\left(-\frac{\gamma^{2}}{8Q\rho^{2}}\right)\eta I_{0}\left(\gamma \right)\ln\left(\exp\left(\gamma \right)\right)d\gamma ,$$

III:

(A19) $$\int_{0}^{\infty }f\left(\gamma \right)\eta I_{0}\left(\gamma \right)\ln\left(\frac{1}{\sqrt{\gamma }}\right)d\gamma =\int_{0}^{\infty }\frac{\gamma }{4Q\rho^{2}}\exp\left(-\frac{\gamma^{2}}{8Q\rho^{2}}\right)\eta I_{0}\left(\gamma \right)\ln\left(\frac{1}{\sqrt{\gamma }}\right)d\gamma .$$

According to the derivation process in Appendix B, I and II can be approximated as

(A20) $$I=\eta \ln\left(\frac{\eta }{\sqrt{2\pi }}\right)\exp\left(2Q\rho^{2}\right),$$

(A21) $$\begin{array}{ll} \mathrm{II} & =\frac{\eta }{4Q\rho^{2}}\int_{0}^{\infty }\gamma^{2}\exp\left(-\frac{\gamma^{2}}{8Q\rho^{2}}\right)I_{0}\left(\gamma \right)d\gamma \\ & =\sqrt{2\pi Q}\eta \rho \exp\left(Q\rho^{2}\right)\left[2Q\rho^{2}I_{1}\left(Q\rho^{2}\right)+\left(1+2Q\rho^{2}\right)I_{0}\left(Q\rho^{2}\right)\right]. \end{array}$$

We introduce the first kind of the modified Bessel function, which is expressed as

(A22) $$I_{\alpha }\left(x\right)={\sum_{m=0}^{\infty }\frac{1}{m!\Gamma \left(m+\alpha +1\right)}\left(\frac{x}{2}\right)}^{2m+\alpha }.$$

Therefore, $I_{0}\left(Q\rho^{2}\right)$ and $I_{1}\left(Q\rho^{2}\right)$ in (A21) are expressed as

(A23) $$\begin{array}{ll} I_{0}\left(Q\rho^{2}\right) & =\sum_{m=0}^{\infty }\frac{1}{m!\Gamma \left(m+\alpha +1\right)}{\left(\frac{Q\rho^{2}}{2}\right)}^{2m+\alpha }\\ & \approx \frac{1}{0!\Gamma \left(1\right)}{\left(\frac{Q\rho^{2}}{2}\right)}^{0}+\frac{1}{1!\Gamma \left(2\right)}{\left(\frac{Q\rho^{2}}{2}\right)}^{2}\\ & \approx 1+\frac{{\left(Q\rho^{2}\right)}^{2}}{4}, \end{array}$$

(A24) $$\begin{array}{ll} I_{1}\left(Q\rho^{2}\right) & =\sum_{m=0}^{\infty }\frac{1}{m!\Gamma \left(m+\alpha +1\right)}{\left(\frac{Q\rho^{2}}{2}\right)}^{2m+\alpha }\\ & \approx \frac{1}{0!\Gamma \left(2\right)}{\left(\frac{Q\rho^{2}}{2}\right)}^{1}+\frac{1}{1!\Gamma \left(3\right)}{\left(\frac{Q\rho^{2}}{2}\right)}^{3}\\ & \approx \frac{Q\rho^{2}}{2}+\frac{{\left(Q\rho^{2}\right)}^{3}}{16}. \end{array}$$

Substituting (A23) and (A24) into (A21), we have

(A25) $$\begin{array}{ll} II & \approx \sqrt{2\pi Q}\eta \rho \exp\left(Q\rho^{2}\right)\left[2Q\rho^{2}\left(\frac{Q\rho^{2}}{2}+\frac{{\left(Q\rho^{2}\right)}^{3}}{16}\right)+\left(1+2Q\rho^{2}\right)\left(1+\frac{{\left(Q\rho^{2}\right)}^{2}}{4}\right)\right]\\ & \approx \sqrt{2\pi Q}\eta \rho \exp\left(Q\rho^{2}\right) \end{array}$$

Note that the approximation in (A15) is obtained by omitting higher-order terms, and the approximation becomes more accurate as the SNR decreases.

(A19) is approximately written as

(A26) $$III\approx \eta \int_{0}^{\infty }\frac{\sqrt{\gamma }}{4\sqrt{2\pi }Q\rho^{2}}\exp\left(-\frac{1}{8Q\rho^{2}}{\left(\gamma -4Q\rho^{2}\right)}^{2}+2Q\rho^{2}\right)\ln\left(\frac{1}{\sqrt{\gamma }}\right)d\gamma .$$

Setting $\upsilon =\gamma -4Q\rho^{2}$, (A26) is rewritten as

(A27) $$\begin{array}{l} \int_{-4Q\rho^{2}}^{\infty }\frac{\sqrt{\upsilon +4Q\rho^{2}}}{4\sqrt{2\pi }Q\rho^{2}}\exp\left(-\frac{1}{8Q\rho^{2}}\upsilon^{2}+2Q\rho^{2}\right)\ln\left(\frac{1}{\sqrt{4Q\rho^{2}}}\right)\ln\left(\frac{1}{\sqrt{1+\upsilon /4Q\rho^{2}}}\right)d\upsilon \\ =\exp\left(2Q\rho^{2}\right)\ln\left(\frac{1}{\sqrt{4Q\rho^{2}}}\right)\int_{-4Q\rho^{2}}^{\infty }\frac{\sqrt{\upsilon +4Q\rho^{2}}}{4\sqrt{2\pi }Q\rho^{2}}\exp\left(-\frac{1}{8Q\rho^{2}}\upsilon^{2}\right)d\upsilon \\ \;\;+\int_{-4Q\rho^{2}}^{\infty }\frac{\sqrt{\upsilon +4Q\rho^{2}}}{4\sqrt{2\pi }Q\rho^{2}}\exp\left(-\frac{1}{8Q\rho^{2}}\upsilon^{2}+2Q\rho^{2}\right)\ln\left(\frac{1}{\sqrt{1+\upsilon /4Q\rho^{2}}}\right)d\upsilon . \end{array}$$

Through numerical simulation, we have

(A28) $$\begin{array}{l} \exp\left(2Q\rho^{2}\right)\ln\left(\frac{1}{\sqrt{4Q\rho^{2}}}\right)\int_{-4Q\rho^{2}}^{\infty }\frac{\sqrt{\upsilon +4Q\rho^{2}}}{4\sqrt{2\pi }Q\rho^{2}}\exp\left(-\frac{1}{8Q\rho^{2}}\upsilon^{2}\right)d\upsilon \approx \exp\left(2Q\rho^{2}\right)\ln\left(\frac{1}{\sqrt{4Q\rho^{2}}}\right), \end{array}$$

(A29) $$\int_{-4Q\rho^{2}}^{\infty }\frac{\sqrt{\upsilon +4Q\rho^{2}}}{4\sqrt{2\pi }Q\rho^{2}}\exp\left(-\frac{1}{8Q\rho^{2}}\upsilon^{2}+2Q\rho^{2}\right)\ln\left(\frac{1}{\sqrt{1+\upsilon /4Q\rho^{2}}}\right)d\upsilon \approx 0.$$

Thus, (A19) is approximated as

(A30) $$III\approx -\eta \exp\left(2Q\rho^{2}\right)\ln\left(\sqrt{4Q\rho^{2}}\right).$$

Based on (A20), (A25), and (A30), (A15) is approximately calculated as

(A31) $$\begin{array}{ll} H_{n} & =-\left|\Theta \right|\left|\Xi \right|\left[\eta \ln\left(\frac{\eta }{\sqrt{2\pi }}\right)\exp\left(2Q\rho^{2}\right)\right.\left.+\sqrt{2\pi Q}\eta \rho \exp\left(Q\rho^{2}\right)-\eta \exp\left(2Q\rho^{2}\right)\ln\left(\sqrt{4Q\rho^{2}}\right)\right]\\ & \approx \ln\left(\frac{2\sqrt{2\pi Q\rho^{2}}\left|\Theta \right|\left|\Xi \right|}{\exp\left(\sqrt{2\pi Q\rho^{2}}-2Q\rho^{2}\right)}\right) \end{array}$$

## Appendix D

In the high-SNR region, the joint *a posteriori* PDF is written as

(A32) $$\begin{array}{ll} p\left(\theta ,\vartheta \left|z\right.\right) & =\frac{V_{S}}{R_{S}}=\eta I_{0}\left(\frac{2\alpha }{N_{0}}\left|G\left(\vartheta ,\theta \right)+F\left(\vartheta ,\theta ,N\right)\right|\right)\\ & =\eta I_{0}\left(\left|\frac{2\alpha }{N_{0}}G\left(\vartheta ,\theta \right)+h_{n}\left(\vartheta ,\theta \right)\right|\right). \end{array}$$

Furthermore, expanding the real and imaginary parts of $h_{n}\left(\vartheta ,\theta \right)$, we have

(A33) $$\begin{array}{ll} h_{n}\left(\vartheta ,\theta \right) & =\varsigma +j\zeta \\ & \approx \varsigma_{0}+\varsigma_{\vartheta }^{\prime }\left(\vartheta -\vartheta_{0}\right)+\varsigma_{\theta }^{\prime }\left(\theta -\theta_{0}\right)+j\left(\zeta_{0}+\zeta_{\vartheta }^{\prime }\left(\vartheta -\vartheta_{0}\right)+\zeta_{\theta }^{\prime }\left(\theta -\theta_{0}\right)\right), \end{array}$$

where the expectation of the variables is all zero. And

(A34) $$E\left({\varsigma_{\vartheta }^{\prime }}^{2}\right)=E\left({\zeta_{\vartheta }^{\prime }}^{2}\right)=\frac{\partial {h_{n}}^{H}}{\partial \vartheta }\frac{\partial h_{n}}{\partial \vartheta }=\frac{L^{2}\left(Q^{3}-Q\right)\sin^{2}\theta_{0}}{2\rho^{2}},$$

(A35) $$E\left({\varsigma_{\theta }^{\prime }}^{2}\right)=E\left({\zeta_{\theta }^{\prime }}^{2}\right)=\frac{\partial {h_{n}}^{H}}{\partial \theta }\frac{\partial h_{n}}{\partial \theta }=\frac{\beta^{2}\left(Q^{3}-Q\right)\cos{\vartheta_{0}}^{2}}{2\rho^{2}}.$$

Therefore, the content in the bracket of the Bessel function in (A32) can be expressed as

(A36) $$\begin{array}{l} \left|\frac{2\alpha }{N_{0}}G\left(\vartheta ,\theta \right)+h_{n}\left(\vartheta ,\theta \right)\right|\\ \approx 2\rho^{2}\left[2Q+\varsigma_{0}-\frac{1}{2}\left(Q^{3}-Q\right)L^{2}\sin^{2}\theta_{0}{\left(\vartheta -\vartheta_{0}-\frac{{\varsigma_{\vartheta }}^{\prime }}{\left(Q^{3}-Q\right)L^{2}\sin^{2}\theta_{0}}\right)}^{2}\right.-\frac{1}{2}\left(Q^{3}-Q\right)L^{2}\cos^{2}\theta_{0}{\left(\theta -\theta_{0}-\frac{{\varsigma_{\theta }}^{\prime }}{\left(Q^{3}-Q\right)L^{2}\cos^{2}\vartheta_{0}}\right)}^{2}\\ \;\;\;\left.+\frac{{\varsigma_{\vartheta }^{\prime }}^{2}+{\zeta_{\vartheta }^{\prime }}^{2}}{2\left(Q^{3}-Q\right)L^{2}\sin^{2}\theta_{0}}+\frac{{\varsigma_{\theta }^{\prime }}^{2}+{\zeta_{\theta }^{\prime }}^{2}}{2\left(Q^{3}-Q\right)L^{2}\cos^{2}\vartheta_{0}}\right]\\ =4Q\rho^{2}+\chi -\left(Q^{3}-Q\right)\rho^{2}L^{2}\sin^{2}\theta_{0}{\left(\vartheta -\vartheta_{m}\right)}^{2}-\left(Q^{3}-Q\right)\rho^{2}L^{2}\cos^{2}\vartheta_{0}{\left(\theta -\theta_{m}\right)}^{2}, \end{array}$$

where

(A37) $$\chi =2\rho^{2}\left(\varsigma_{0}+\frac{{\varsigma_{\vartheta }^{\prime }}^{2}+{\zeta_{\vartheta }^{\prime }}^{2}}{2\left(Q^{3}-Q\right)L^{2}\sin^{2}\theta_{0}}+\frac{{\varsigma_{\theta }^{\prime }}^{2}+{\zeta_{\theta }^{\prime }}^{2}}{2\left(Q^{3}-Q\right)L^{2}\cos^{2}\vartheta_{0}}\right),$$

And $E\left(\chi \right)=1$.

Substituting (A19) into (A15), we can rewrite the PDF as

(A38) $$\begin{array}{l} p\left(\theta ,\vartheta \left|z\right.\right)\\ \approx \eta I_{0}\left[4Q\rho^{2}+\chi -\left(Q^{3}-Q\right)\rho^{2}L^{2}\sin^{2}\theta_{0}{\left(\vartheta -\vartheta_{0}\right)}^{2}\right.\left.-\left(Q^{3}-Q\right)\rho^{2}L^{2}\cos^{2}\vartheta_{0}{\left(\theta -\theta_{0}\right)}^{2}\right], \end{array}$$

Therefore,

(A39) $$\begin{array}{ll} R_{S} & \approx \iint_{s}p\left(\theta ,\vartheta \left|z\right.\right)d\vartheta d\theta \\ & \approx \iint_{s}I_{0}\left[4Q\rho^{2}+\chi -\left(Q^{3}-Q\right)\rho^{2}L^{2}\sin^{2}\theta_{0}{\left(\vartheta -\vartheta_{0}\right)}^{2}\right.\left.-\left(Q^{3}-Q\right)\rho^{2}L^{2}\cos^{2}\vartheta_{0}{\left(\theta -\theta_{0}\right)}^{2}\right]d\vartheta d\theta . \end{array}$$

According to (A5), (A39) is further written as

(A40) $$\begin{array}{ll} R_{S} & \approx \iint_{s}\frac{\exp\left[4Q\rho^{2}+1-\left(Q^{3}-Q\right)\rho^{2}L^{2}\sin^{2}\theta_{0}{\left(\vartheta -\vartheta_{0}\right)}^{2}\left.-\left(Q^{3}-Q\right)\rho^{2}L^{2}\cos^{2}\theta_{0}{\left(\theta -\theta_{0}\right)}^{2}\right]\right.}{\sqrt{2\pi \left[4Q\rho^{2}+1-\left(Q^{3}-Q\right)\rho^{2}L^{2}\sin^{2}\theta_{0}{\left(\vartheta -\vartheta_{0}\right)}^{2}\left.-\left(Q^{3}-Q\right)\rho^{2}L^{2}\cos^{2}\theta_{0}{\left(\theta -\theta_{0}\right)}^{2}\right]\right.}}d\vartheta d\theta \\ & \approx \frac{\exp\left(4Q\rho^{2}+1\right)}{\sqrt{2\pi \left(4Q\rho^{2}+1\right)}}\iint_{s}\exp\left[-\left(Q^{3}-Q\right)\rho^{2}L^{2}\sin^{2}\theta_{0}{\left(\vartheta -\vartheta_{0}\right)}^{2}\left.-\left(Q^{3}-Q\right)\rho^{2}L^{2}\cos^{2}\vartheta_{0}{\left(\theta -\theta_{0}\right)}^{2}\right]\right.d\vartheta d\theta . \end{array}$$

Then, using the integral formula of the Gaussian distribution, we have

(A41) $$R_{S}\approx \frac{\sqrt{\pi }\exp\left(4Q\rho^{2}+1\right)}{\sqrt{2\left(4Q\rho^{2}+1\right)}\left(Q^{3}-Q\right)\rho^{2}L^{2}\sin\theta_{0}\cos\vartheta_{0}}.$$

## Appendix E

Appendix E gives the specific derivation process of the CRB in (54) and (55). The steering vector matrix in (6) is written as

(A42) $$A\left(\vartheta ,\theta \right)=\left[\begin{array}{c} 1,\\ e^{\frac{j2\pi d\cos\vartheta \sin\theta }{\lambda }}\\ \vdots \\ e^{\frac{j2\pi (Q-1)d\cos\vartheta \sin\theta }{\lambda }}\\ 1\\ e^{\frac{j2\pi d\sin\vartheta \sin\theta }{\lambda }}\\ \vdots \\ e^{\frac{j2\pi (Q-1)d\sin\vartheta \sin\theta }{\lambda }} \end{array}\right].$$

Then,

(A43) $$\frac{\partial A\left(\vartheta ,\theta \right)}{\partial \vartheta }=j\frac{2\pi d}{\lambda }\left[\begin{array}{c} 0\\ -\sin\vartheta \sin\theta \\ \vdots \\ -(Q-1)\sin\vartheta \sin\theta \\ 0\\ \cos\vartheta \sin\theta \\ \vdots \\ (Q-1)\cos\vartheta \sin\theta \end{array}\right]⊙A\left(\vartheta ,\theta \right),$$

(A44) $$\frac{\partial A\left(\vartheta ,\theta \right)}{\partial \theta }=j\frac{2\pi d}{\lambda }\cos\theta \left[\begin{array}{c} 0\\ \cos\vartheta \\ \vdots \\ \left(Q-1\right)\cos\vartheta \\ 1\\ \sin\vartheta \\ \vdots \\ \left(Q-1\right)\sin\vartheta \end{array}\right]⊙A\left(\vartheta ,\theta \right).$$

where ⊙ represents the Hadamard product.

Substituting (A43) and (A44) into (51) and (52),

(A45) Fϑ,ϑ=2ρ2Re∂Aϑ,θ∂ϑH∂Aϑ,θ∂ϑ=2ρ2π2d23λ2QQ−1Q+1sin2θ=2Qρ2L2sin2θ.

(A46) Fϑ,θ=2ρ2Re∂Aϑ,θ∂ϑH∂Aϑ,θ∂θ=0.

(A47) Fθ,θ=2ρ2Re∂Aϑ,θ∂ϑH∂Aϑ,θ∂ϑ=2ρ2π2d23λ2QQ−1Q+1cos2ϑ=2Qρ2L2cos2ϑ.

Since azimuth and elevation DOAs are independent of each other, the CRB for azimuth and elevation DOA joint estimation can be expressed as

(A48) $$\mathrm{CRB}={\mathrm{CRB}}_{\vartheta ,\vartheta }•{\mathrm{CRB}}_{\theta ,\theta }={\left(4Q^{2}\rho^{4}L^{4}\sin^{2}\theta \cos^{2}\vartheta \right)}^{-1}.$$
